# Relation-Aware Heterogeneous Graph Learning for miRNA–Drug Response Association Prediction Under Cold-Start Generalization

**DOI:** 10.3390/ijms27209013

**Published:** 2026-10-11

**Authors:** Murtada K. Elbashir

**Affiliations:** Department of Information Systems, College of Computer and Information Sciences, Jouf University, Sakaka 72441, Saudi Arabia; mkelfaki@ju.edu.sa

**Keywords:** miRNA–drug response association, heterogeneous graph learning, cancer drug resistance, cold-start prediction

## Abstract

(1) Background: Cancer drug resistance can be influenced by microRNAs (miRNAs), which can alter gene expression and cellular pathways that determine how cancer cells respond to treatment. Potential miRNA–drug response associations can be identified using computational models; however, evaluating these models using edge-based evaluation can result in an optimistic view of model generalization since test miRNAs or drugs can appear in other response associations during training. (2) Methods: This paper presents a relation-aware heterogeneous learning framework that integrates miRNA expression profiles, miRNA–gene regulatory interactions, gene–gene functional associations, drug molecular features, and distinct drug–gene relations in a unified miRNA–gene–drug network. A Heterogeneous Graph Transformer (HGT) was used to preserve relation-specific information, and biological paths connecting each miRNA to each drug were used as additional information for the corresponding candidate miRNA–drug pair. A leakage-safe five-fold cross-validation approach was used to evaluate the proposed framework under different settings: edge, miRNA-cold, drug-cold, and double-cold. (3) Results: The proposed framework obtained an AUC and AUPR of 0.9537 ± 0.0044 and 0.9494 ± 0.0069, respectively, under the edge-based setting. The performance remained high under the miRNA-cold-based setting, where the model achieved an AUC and AUPR of 0.9466 ± 0.0061 and 0.9436 ± 0.0049, respectively. A substantial drop in performance was observed under the drug-cold setting, where the proposed framework obtained an AUC and AUPR of 0.6685 ± 0.0354 and 0.6534 ± 0.0569, respectively. The performance was also substantially reduced under the double-cold-based setting. The results also show that the structural resemblance alone is not enough to explain generalization to new drugs, since no significant relationship between cold-start performance and either ChemBERTa molecular similarity or DrugBank target similarity was shown by drug-level analysis. Literature evidence supported 6 out of 20 top-ranked previously unlabeled predictions compared to 1 of 120 randomly selected controls (Fisher’s exact test, *p* = 2.63 × 10^−5^; empirical *p* = 2.80 × 10^−5^). (4) Conclusions: The obtained results show that the proposed framework can rank candidate miRNA–drug response associations effectively. They also show that predicting response associations under the drug-cold-based setting remains much harder than predicting for similarly held-out miRNAs, which is an important limitation of conventional edge-based evaluation.

## 1. Introduction

The treatment of malignancy is often affected by drug resistance and related mechanisms. Even drugs that initially were effective in malignancy treatment can lose their effectiveness over time. Resistance can develop through various mechanisms, including altered uptake and efflux of drugs, modifications of drug targets, enhancement of DNA repair, drug metabolism alterations, and activation of alternate cell survival pathways [1,2]. These mechanisms seldom operate in isolation. They are part of regulatory networks that can affect how cells respond to treatment, making the identification of molecular factors that influence drug sensitivity an important challenge in precision medicine. MicroRNAs (miRNAs) are non-coding RNAs that regulate post-transcriptional gene expression and therefore can influence many genes involved in different biological processes [3,4]. They are particularly relevant to the specification of drug response. Altering levels of expression of miRNA can affect apoptosis, cell-cycle regulation, epithelial–mesenchymal transition, DNA-damage repair, drug transport, and signaling pathways that promote cell survival [5,6]. Depending on the genes it regulates, an miRNA may increase drug resistance in one cellular context but increase drug sensitivity in another. Experimental studies have reported many miRNA-related drug responses, and these findings have also been collected in curated databases. For example, ncRNADrug reports 29,551 experimentally validated ncRNA–drug response entries involving 266 drugs and 2248 miRNAs [7]. Even with this amount of evidence, only a small fraction of all possible miRNA–drug combinations has been experimentally characterized. Experimental associations may need expression profiling followed by targeted perturbation, quantitative PCR, reporter assays, protein level measurements, and drug-sensitivity experiments. Such processes can only be carried out on a small number of candidate miRNA–drug pairs and are hard to scale up. Computational prediction can help to reduce this search space by ranking associations for further experimental analysis.

The majority of the initial computational models have approached the problem of miR-NA–drug resistance association prediction as an association inference problem. One of the few methods that specifically addresses this prediction task as a link prediction problem on attributed bipartite graphs is GCMDR. GCMDR employed graph convolutions to combine resistance associations, miRNA expression profiles with the structural fingerprints of the drugs and functional data. It has been demonstrated by the performance of GCMDR that the learning of molecular properties and of graph structure is not carried out separately (as similarity measures) but can be performed simultaneously [8]. Another study, NASMDR, applies graph isomorphism networks and neural architecture search, but instead of using the miRNA-drug graph relies only on molecular and sequence attributes. Thus, this model does not depend so much on the association density and uses learned sequence representation for miRNAs [9]. Subsequent works focused on specific challenges posed by sparse and noisy association networks. For example, GAMMDR uses a graph autoencoder with path-masking that performs miRNA–drug graph reconstruction with the suppression of the possible effect of noise in the graph data [10]. Some other approaches have tried to increase the robustness of the representations with the help of sparse supervision via contrastive learning and alternative graph views. All those works have helped to enhance the representation of miRNAs and drugs; however, most of the approaches still treat the resistance prediction as the reconstruction of the association network. A more recent direction of research considers the miRNA–drug bipartite graph as a starting point, and utilizes biological intermediates in the process. It is a substantial step forward because the miRNAs do not influence drug resistance through network interactions but through the genes and the drugs interact with the proteins associated with pharmacological actions, transportation, metabolism, and disposition. MPHGNN creates a heterogeneous miRNA–gene–drug network that incorporates miRNA expression profiles, drug structural descriptors, gene data and metapath learning [11]. The network has two branches representing biological pathways and network structure via metapath and global branches. The recent research, iNcRD-HG, extends this approach to the more general ncRNA–drug prediction problem by using the context-enriched heterogeneous network and relation-aware message passing. The authors have especially focused on the limitations of the previous formulations—the semantic distortion in sparse bipartite networks and the loss of relational meaning [12].

Due to these developments, heterogeneous graph learning becomes a natural framework for the prediction of miRNA–drug response associations. However, retaining the biological meaning of the relation is important to make heterogeneity useful. The relationship between a drug and a gene can be a target, enzyme, or transporter/carrier relationship. These interactions represent various pharmacologic pathway interactions and should not be reduced to a generic drug–gene edge interaction. DrugBank clearly identifies these relationships and offers curated drug, target, enzyme, transporter, carrier, structural and pharmacological data [13]. Similarly, miRNA–gene regulation and gene–gene functional association are two different types of biological processes. The contribution of each relation type to an miRNA–drug association can be learnt by a model that retains these relations instead of considering all neighboring nodes as biologically equivalent.

A suitable mechanism in this setting is provided by Heterogeneous Graph Transformers (HGTs). In HGT, the attention and message-passing operations are based on node- and edge-type-dependent parameters, which enable the model to transform information following the semantic type of the source node, the target node, and the connected relation [14]. This relation-aware formulation is suitable for an miRNA–gene–drug network where the meaning of the regulatory, functional or pharmacological edges is different. Another complementary source of information is the molecular structure of the drug. Some molecular encoders based on transformers (like ChemBERTa) are trained directly from the SMILES string on a large-scale self-supervised learning task [15] and can thus learn representations intrinsic to the chemical structure without relying on known response-association labels. This can be particularly useful if a drug used by the model is different from the ones used for response-association supervision in the training process. This results in an under-studied issue in the field of model architecture, namely, how to assess the performance of the predictive models. A large majority of studies on the relationship between miRNAs and drug resistance have conducted cross-validation by randomly dividing individual associations between miRNAs and drug resistance into training and testing sets. A model can be evaluated whether it can reconstruct missing association between entities that are already present in the other parts of the training data through this kind of edgewise setting. Although it is a valid network-completion task, it does not test whether this model can be used to predict a new miRNA or drug that has no response associations in the training set. The distinction matters because the two settings evaluate different forms of generalization. A random edge split would mean that the test drug could already be linked to several training miRNAs, which is not the real prediction for an unseen drug.

Cold-start prediction at the entity level is considerably more challenging. For miRNA-cold prediction, all response associations involving the held-out miRNAs are excluded from training. A similar restriction applies to held-out drugs for drug-cold prediction. Double-cold evaluation goes further by the necessity of both members of the test pair to be hidden with respect to response-association labels. Different forms of generalization are tested by these settings and identical behavior is not expected to be produced by them. Even if miRNA is unseen, its expression profile and regulatory connections with genes can still be determined. In contrast, an unseen drug cannot be placed by relying on its response-association history but rather must be placed based on intrinsic chemical information and auxiliary pharmacological relationships. Leakage is a second methodological question that arises when considering cold-start evaluation. In the case of an miRNA–drug response-association edge that is part of validation or test data, the model may indirectly learn the relationship it aims to predict if the edge is permitted to be part of the graph message passing. The prediction target must then be isolated from the permanent biological context; otherwise, a strict evaluation is not possible. Only training response-association edges are allowed to be included in the fold-specific message passing graph; the validation and test target edges are excluded. This is even more crucial in entity-cold settings where all response associations that involve a held-out entity should not be present during training process. In this study, this separation is made explicit in a new experimental design.

The bigger question is what convincing validation is in general. Cross-validated AUC or AUPR is an indication of predictive discrimination against the selected benchmark but does not necessarily indicate high-confidence predictions in terms of biologically meaningful relationships outside training labels. Independent evidence is thus beneficial. A list of previously unlabeled, high-confidence predictions that were already made can be searched in the literature and compared against a list of unlabeled, same-evidence predictions. This does not mean it can act as a substitute for the lab’s prospective experimental validation, but it can be an external check that the model can rank associations that have independent experimental support properly. This distinction is evident from the currently available databases, where experimentally confirmed ncRNA–drug relationships are highly context-dependent and the same ncRNA can exhibit various drug-response effects in different diseases or cell lines [7].

In this work, a relation-aware heterogeneous learning framework based on the Heterogeneous Graph Transformer (HGT) is presented for predicting miRNA–drug response associations. The framework combines miRNA expression profiles, miRNA–gene regulatory relations, gene–gene functional relations, molecular representations of drugs and pharmacologically distinct DrugBank drug–gene relations into a single heterogeneous graph. The suggested methodological design solves three limitations pertaining to heterogeneous miRNA–drug prediction. First, relation-aware modeling preserves the meaning of the different drug–gene interactions, including target, enzyme, transporter, and carrier relations, rather than treating them all as a single generic drug–gene relation. Second, pair-specific path attention tackles the issue that multiple biological paths can link the same miRNA–drug pair but may not make the same contribution to a particular prediction; the multiple paths for a given pair are therefore weighted using their candidate-pair representation. Third, adaptive fusion avoids assigning a fixed contribution to the global HGT representation and the pair-specific path representation. Rather, the relative contribution is learned for each pair of candidates, with the global representation being used if a valid biological path is not available.

In addition to these architectural elements, this study provides a general contribution in three areas. First, it offers a unified heterogeneous semantic context for pairs, preserving biological and pharmacological relation semantics while introducing pair-specific biological context. Second, the model is evaluated for generalization by testing it using leakage-safe edge, miRNA-cold, drug-cold, and double-cold protocols and full-candidate ranking analysis, without the presence of either miRNAs or drugs in the response-association supervision. Third, this study explores the impact of model components and drug-level factors on cold-start performance and evaluates high-confidence model predictions for previously unlabeled drugs using independent literature evidence and randomly selected unlabeled controls.

## 2. Results and Discussion

### 2.1. Overall Performance Under Edge-Based Evaluation

The predictive ability of the proposed model was initially tested in the conventional edge-based setting, where the association of each miRNA–drug response was excluded from the training set, but the corresponding miRNAs and drugs were present in the other training associations. This setting is used to assess the initial ability of the model to recover previously unseen relationships between different types of entities in the heterogeneous network. As shown in Table 1, the outer folds were consistently well classified by the proposed model using the edge-based evaluation. An AUC of 0.9537 ± 0.0044 with a 95% CI ranging from 0.9499 to 0.9576 and a mean AUPR of 0.9494 ± 0.0069 with a 95% CI ranging from 0.9433 to 0.9554 were obtained by the model. Also, the variation between the folds is small, indicating that a single favorable data partition was not the cause of the observed discrimination. The model also achieved an MCC of 0.7806 ± 0.0095, Brier score of 0.0834 ± 0.0017, and ECE of 0.0354 ± 0.0034, indicating reasonably calibrated probability estimates. The results demonstrate the good predictive performance provided by the heterogeneous graph and pair-specific path representations. However, this performance does not imply that it can be extended to previously unseen miRNAs or drugs, as the edge-based setting alone does not establish this.

**Figure 1:**
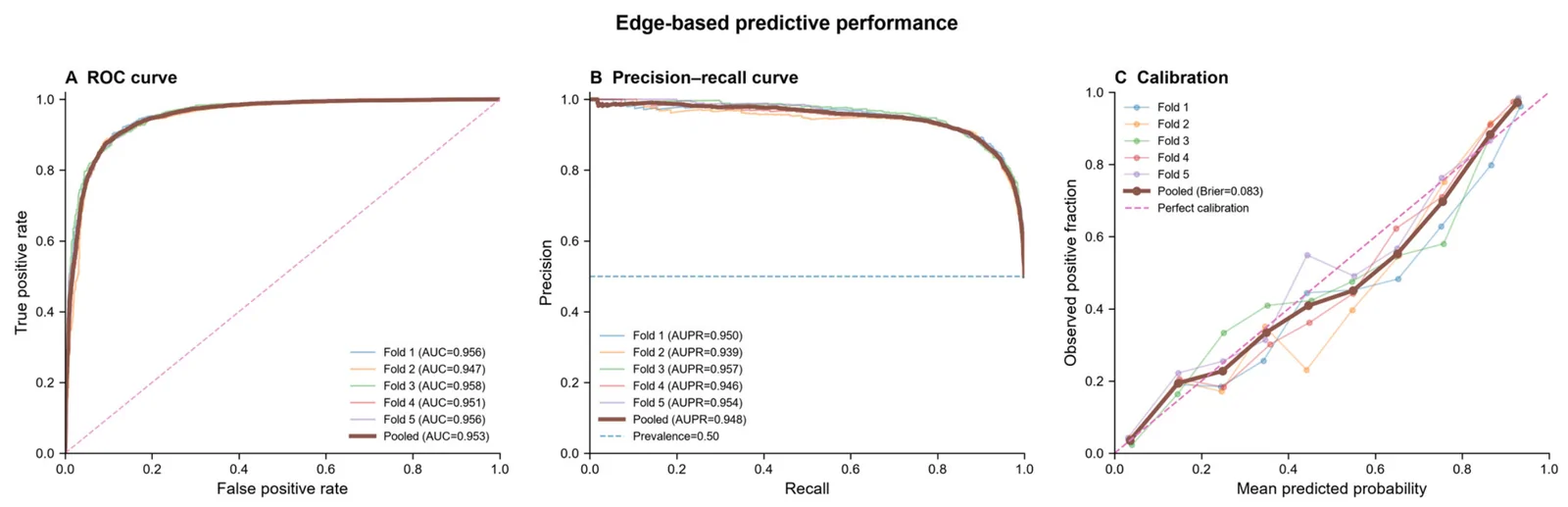
Predictive performance under edge-based evaluation. (**A**) Receiver operating characteristic (ROC) curves. (**B**) Precision–recall (PR) curves. (**C**) Reliability diagram, representing the fraction of positive associations at the outer folds compared with the predicted probabilities. The solid curves (pooled) are used to represent variation.

The predictive behavior of the model throughout the five outer folds is depicted in Figure 1. The concentration of the ROC curves in the upper-left corner (Figure 1A) and AUC values between 0.947 and 0.958 with a pooled AUC of 0.953 show that the model has good performance. The model maintained discrimination that is consistent across the five-fold partition, and this is evident from the minimal fluctuation between folds. This is in line with the results shown in Table 1. The AUPRs of precision-recall curves (Figure 1B) ranged from 0.939 to 0.957, and the pooled AUPR value was 0.948. The precision-recall curves remained above the prevalence line of the positive class most of the time over the recall range, suggesting that the model has a high precision rate while capturing many positive miRNA-drug response relations. Figure 1C presents the reliability plot of the model with a pooled Brier Score of 0.083. Its pooled calibration plot was consistent with the ideal diagonal. The discrimination ability was relatively consistent across folds, in contrast to the calibration ability. This is reflected in the small deviations seen in some folds, particularly at intermediate predicted probabilities.

### 2.2. Generalization Under Cold-Start Evaluation

The proposed model was analyzed for three cold-start scenarios: miRNA-cold, drug-cold, and double-cold to see if the performance reported in edge-based evaluation could be obtained for unseen entities. The model design and hyperparameters were not changed in the three cold-start settings. Only the data partition strategy was changed so that the test entities were not taken into account as response-association for training. Table 2 shows the results obtained in the three cold-start settings. In miRNA-cold, our model attained an accuracy of 0.8812 ± 0.0095, F1 of 0.8829 ± 0.0095, and MCC of 0.7631 ± 0.0190. The AUC and AUPR are 0.9466 ± 0.0061 and 0.9436 ± 0.0049, respectively. These results indicate that the model can be generalized to miRNAs that are not considered in the training associations. In contrast, performance was lower when drugs were not used as response-association supervision. In the drug-cold setting, the accuracy, F1 score, and MCC are 0.6049 ± 0.0606, 0.4699 ± 0.1884, and 0.2305 ± 0.1275, respectively. The AUC and AUPR are also reduced to 0.6685 ± 0.0354 and 0.6534 ± 0.0569. The recall values between folds were highly variable with respect to response-association supervision, showing less stability in identifying the positive associations when drugs were unseen. The results from Table 2 show that the more challenging setting is the double-cold setting, where the training associations do not contain the miRNAs and the drugs that are present in the test set. Overall, the results from Table 2 show the difference between miRNA-side and drug-side generalization. The model performance substantially worsened when drugs were unseen, and the double-cold setting also had poorer performance compared to the edge-based and miRNA-cold settings. This raises the question of what drug-related information helps the model make predictions for previously unseen compounds, and this needs to be examined further.

**Figure 2:**
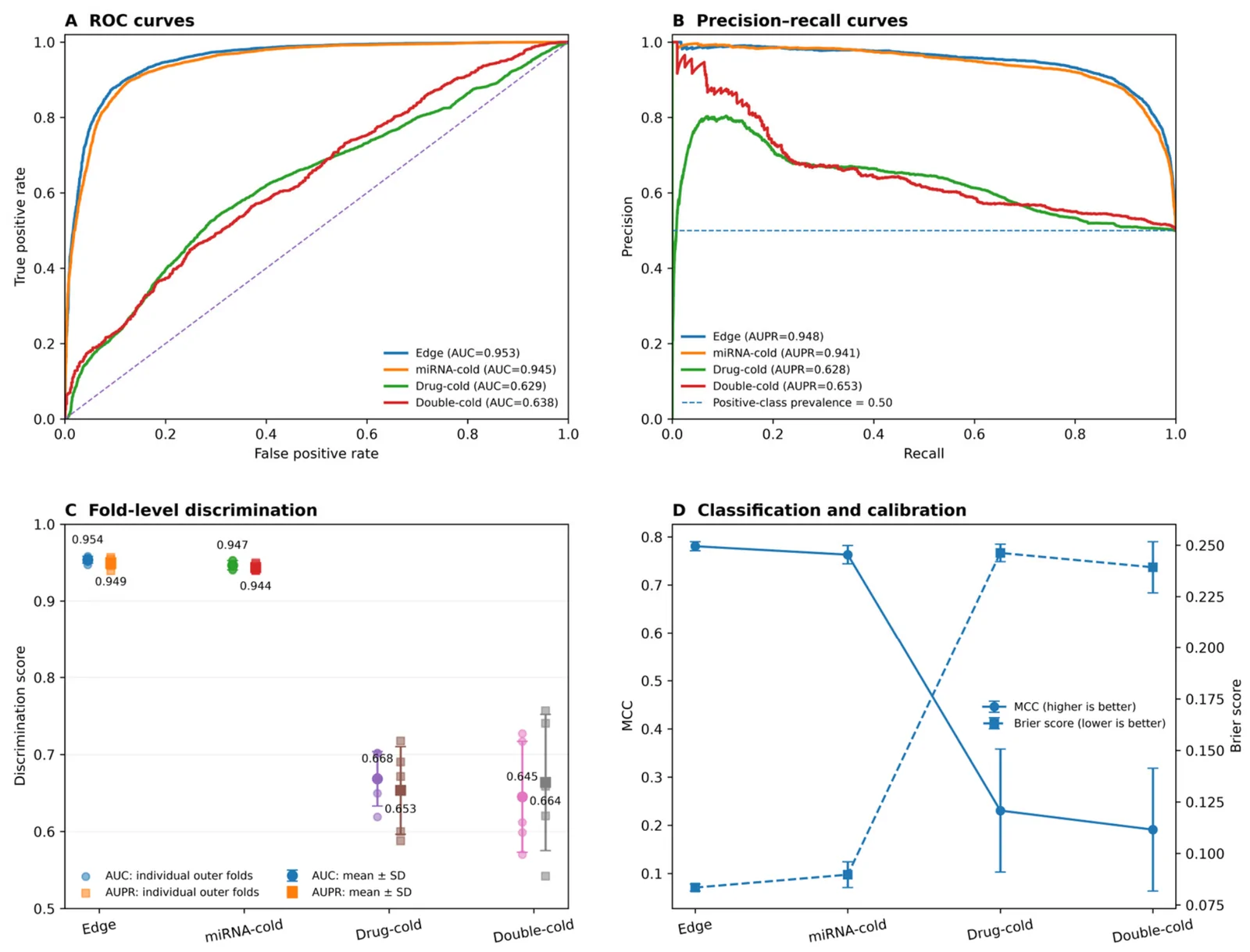
Predictive performance across edge-based and cold-start settings. (**A**) Pooled ROC and (**B**) PR curves for edge, miRNA-cold, drug-cold, and double-cold evaluation. (**C**) Fold-level AUC and AUPR (mean ± SD). (**D**) Fold-level MCC and Brier score. Solid lines represent MCC (left y-axis), while dashed lines represent the Brier score (right y-axis).

Figure 2 shows the model performance under the more restrictive evaluation. The ROC and PR curves are similar for the edge and miRNA-cold evaluations. The pooled AUC decreased slightly from edge-based evaluation to miRNA-cold evaluation; the results were 0.9527 and 0.9451, respectively. The pooled AUPR has also decreased from 0.9477 to 0.9411. In addition to the fold-level results, the model still has most of its predictive power when it is tested on unseen miRNAs. When we introduce unseen drugs, the behavior is completely different. The pooled AUC and the AUPR dropped to 0.6294 and 0.6284, respectively. Similar behavior was observed for the double-cold setting, with the pooled AUC and the AUPR at 0.6379 and 0.6529, respectively. Given the much larger drop in the two different settings, we can see that generalization becomes more difficult when drugs are removed from the training associations. As shown by the similar performance for both drug-cold and double-cold testing, unseen drugs are likely to account for the vast majority of the difficulty in the double-cold setting. This is clearly reflected in the fold-level results in Figure 2C. AUC and AUPR were still high on edge and miRNA-cold folds but substantially lower on drug-cold and double-cold. There was also more variation between folds, particularly on double-cold. Thus, the drop in performance is not only for pooled predictions but also for different held-out partitions.

A different view of the change is given by MCC and Brier scores (Figure 2D). For the evaluation under edge and miRNA-cold conditions, MCC was high, but it dropped drastically when drugs were not seen. The Brier score, which measures the mean squared difference between predicted probabilities and observed outcomes, was about 0.08–0.09 for edge and miRNA-cold evaluation but rose to 0.24–0.25 for drug-cold and double-cold evaluations. Therefore, both the classification accuracy and the calibration of the predicted probabilities were lower for unseen drugs. In general, the results shown in Figure 2 confirm the differences in cold start behavior shown in Table 2. The proposed model generalizes well to unseen miRNAs but not as well for unseen drugs. This difference indicates that drug information is also one of the key factors affecting cold-start generalization. Although the results show reduced performance in the drug-cold setting, they do not explain why the model predicts some unseen drugs more accurately than others. This is studied in the drug-level analysis of the factors related to cold-start performance in the next section.

### 2.3. Full-Candidate Ranking Under Cold-Start Evaluation

In addition to the sampled-unlabeled classification analysis, the model was also tested by ranking all of the eligible candidate pairs in each cold start test fold. In the miRNA-cold setting, each held-out miRNA was ranked against all 216 drugs. In the drug-cold setting, each held-out drug was ranked against all 635 miRNAs. In the double-cold setting, only the combination of the held-out miRNAs and held-out drugs was ranked, and the performance in miRNA-drug and drug-to-miRNA directions was evaluated. For the double-cold evaluation, we ranked the candidate pairs by the mean among the three seeds of the sigmoid-transformed raw logits. This value is used as a model-generated association score, not as a biological probability.

The miRNA-side and drug-side cold-start generalization was different for the full candidate ranking of the model. For miRNA-cold evaluation (see Table 3), the model achieved an MRR of 0.8044 ± 0.0389 and MAP of 0.5462 ± 0.0216. Hits@10 and Hits@20 were 0.9669 ± 0.0131 and 0.9917 ± 0.0058, respectively, while Recall@10 and Recall@20 were 0.5861 ± 0.0160 and 0.8272 ± 0.0110. In the drug-cold assessment, the model achieved an MRR of 0.2343 ± 0.0282 and MAP of 0.1348 ± 0.0149. Hits@10 and Hits@20 were 0.4162 ± 0.0648 and 0.5228 ± 0.0275, with corresponding Recall@10 and Recall@20 of 0.1267 ± 0.0407 and 0.2121 ± 0.0400, respectively.

In the double-cold case, drug-to-miRNA ranking gave an MRR of 0.3533 ± 0.0328 and MAP of 0.2404 ± 0.0169, while miRNA-to-drug ranking gave an MRR of 0.2400 ± 0.1760 and MAP of 0.1872 ± 0.1213. These results show that the excellent miRNA-cold performance achieved in the sampled classification analysis also holds for ranking in the entire eligible candidate space, but prioritization for unseen drugs is much more challenging. In this way, the full-candidate analysis complements, not replaces, the sampled unlabeled classification benchmark.

### 2.4. Protocol-Aligned Baseline Comparison

To estimate how much of the cold-start performance was due to the proposed architecture, rather than the dataset itself or node features, three protocol-aligned controls were evaluated with the same data partitions and candidate construction. We used the same full model for all three protocols. These controls are: (1) training-association degree/popularity baseline, (2) a feature-only MLP with miRNA expression and ChemBERTa drug features, and (3) an HGT-only model without the biological path branch in the pair. For the full model, Table 4 shows the classification values that correspond to the primary evaluation, while the ranking was obtained from the full candidate ranking analysis using the same outer-fold partitions. As can be seen from Table 4, both the full model and HGT-only model had similar results in miRNA-cold evaluation, even though the degree/popularity and feature-only baselines had lower AUC and AUPR. In drug-cold evaluation, however, the degree/popularity baseline had higher AUC and AUPR than the full model. The full model was still better than the feature-only MLP and HGT-only model in AUPR. In the double-cold evaluation, the full model had the highest AUPR among the four models but the feature-only MLP had a similar AUC. Overall, the degree/popularity baseline showed the most uneven ranking pattern among the models, while no single model achieved the highest ranking in both directions of the double-cold setting. The results show that association frequency and intrinsic features can contribute substantially to prediction, and that the full architecture is not always superior to the simpler models in full-candidate ranking.

### 2.5. Drug-Level Determinants of Cold-Start Generalization

Table 5 illustrates the relationships between characteristics of the drug and predictive performance in the drug-cold setting. There were no significant correlations between drug-level AUC and chemical similarity to response-association-supervised training drugs or target similarity. The maximum ChemBERTa similarity value was not significantly correlated with AUC (ρ = 0.015, *p* = 0.8320), nor was the maximum DrugBank target similarity value (ρ = −0.046, *p* = 0.5804). After multiple-testing correction, four measures of biological connectivity showed small negative correlations with AUC. These measures included biological path coverage (ρ = −0.234, q = 0.0092), mean number of biological paths (ρ = −0.232, q = 0.0092), drug–gene relation degree (ρ = −0.181, q = 0.0445) and DrugBank target degree (ρ = −0.166, q = 0.0488). The observed associations with biological connectivity were small and negative and should be interpreted with caution, particularly because different types of associations were observed across drugs.

**Figure 3:**
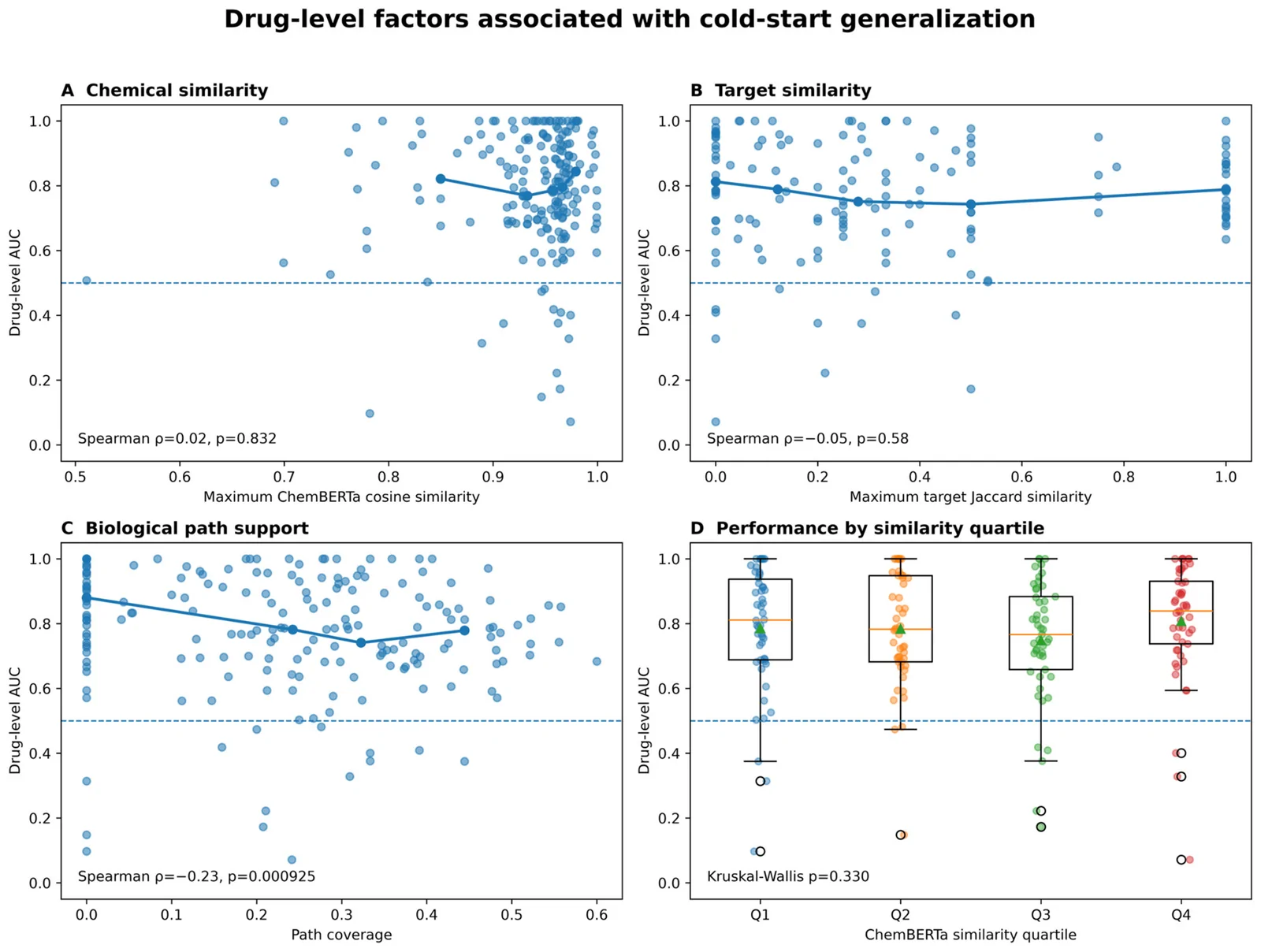
Drug-level factors associated with performance under drug-cold evaluation. (**A**) Drug-level area under the curve (AUC) for each held-out drug versus its maximum ChemBERTa cosine similarity to response-association-supervised training drugs. (**B**) Drug-level AUC versus maximum DrugBank target Jaccard similarity to response-association-supervised training drugs. (**C**) Relationship between drug-level AUC and biological path coverage. Spearman correlations are reported in panels **A**–**C**; the dashed line in panels **A**–**D** marks AUC = 0.5. (**D**) Drug-level AUC across ChemBERTa similarity quartiles. Points represent held-out drugs, boxes show the median and interquartile range, and triangles indicate the mean.

The role of drug-level characteristics and predictive performance in the drug-cold setup is addressed in Figure 3. Drug-level AUC was not significantly related to chemical similarity to response-association-supervised training drugs, as shown in Figure 3 (Spearman ρ = 0.02 and *p* = 0.832). The same was true for DrugBank target similarity (Figure 3), where the relationship with AUC was weak and not significant (ρ = −0.05 and *p* = 0.580). The performance also had no clear difference between the ChemBERTa similarity quartiles (Kruskal–Wallis *p* = 0.330 and Figure 3D). So, unseen drugs that were more chemically similar to the response-association-supervised training drugs did not necessarily have higher predictive performance. Biological path coverage was found to have a small negative correlation with drug-level AUC (ρ = −0.23, *p* = 0.0009; Figure 3C), indicating that drugs with higher path coverage showed lower AUC values in the full drug-level analysis. This association might therefore be interpreted cautiously, as the number of evaluated associations varied for various drugs.

Since the number of evaluated associations varies widely between drugs, we have done a sensitivity analysis to see whether the correlations reported in Table 5 were affected by the drug support. Of the 197 drugs for which drug-level AUC could be obtained, the median number of positive test associations was 2 (IQR 1–13, range 1–446) and the median number of sampled unlabeled pairs was 26 (IQR 18–34, range 6–64). The 19 drug nodes for which there was no positive miRNA-drug response association were not included in the drug-level AUC analysis. The correlation analysis was repeated after requiring at least five positive and five sampled unlabeled pairs for each drug, so we have 71 drugs. There were no significant correlations between AUC and biological path coverage (ρ = −0.155, q = 0.3428), average number of paths (ρ = −0.226, q = 0.2141), degree of drug-to-gene relationship (ρ = −0.209, q = 0.2141), and DrugBank target degree (ρ = −0.232, q = 0.2141).

### 2.6. Ablation Analysis of Model Components

The role of the main components of the proposed architecture was examined in the edge-based setting. All ablation variants were based on the same outer folds, random seeds and hyperparameters, but no variant was retuned. I used the canonical full-model results from the first edge-based evaluation as reference, and then we tested all of our ablation variants on the same outer folds. The removal of individual components leads to very small differences in performance (see Table 6). HGT-only demonstrates marginally higher AUC (0.9553 ± 0.0037) and AUPR (0.9517 ± 0.0066) than full model (0.9537 ± 0.0044 and 0.9494 ± 0.0069) and the accuracy decreased slightly from 0.8900 to 0.8845 and also the MCC decreased marginally from 0.7806 to 0.7728. In this experimental setup, the path branch does not improve the discrimination of predictions as measured by AUC or AUPR but instead leads to marginally higher threshold-based measures.

Changing the fusion method from adaptive to fixed makes no difference to AUC (both models have 0.9537 AUC), while AUPR goes from 0.9494 to 0.9489 and MCC from 0.7806 to 0.7749. Dropping the relation embeddings or HumanNet confidence also does not result in a big difference. Comparison of AUC, AUPR, MCC and Brier score at the pair-fold level with a Wilcoxon test for differences between models, followed by Benjamini-Hochberg correction (All q ≥ 0.25), did not demonstrate any significant difference.

Table 7 shows a separately executed matched cold-start ablation analysis, where the entire model and the corresponding ablation variants have been considered in the same experimental run, so the full model estimates in this table are somewhat different from the estimates reported elsewhere.

Cold-start ablation revealed a much more differentiated pattern than edge-based analysis (Table 7). For drug-cold classification, removing the biological path branch reduces AUPR from 0.6710 to 0.6091, dropping the DrugBank relation types reduces it to 0.6026, and ChemBERTa drug features reduce it to 0.5961. In this setting, fixed fusion produced a similar AUPR of 0.6747 compared with 0.6710 for the full model. With double-cold evaluation, the full model has an AUPR of 0.6591 relative to 0.6194 for HGT only and 0.6361 for relation collapsed and 0.5849 without ChemBERTa features. In other words, the contributions of the individual components were determined by the setting and metric and did not necessarily improve the performance as a whole.

**Figure 4:**
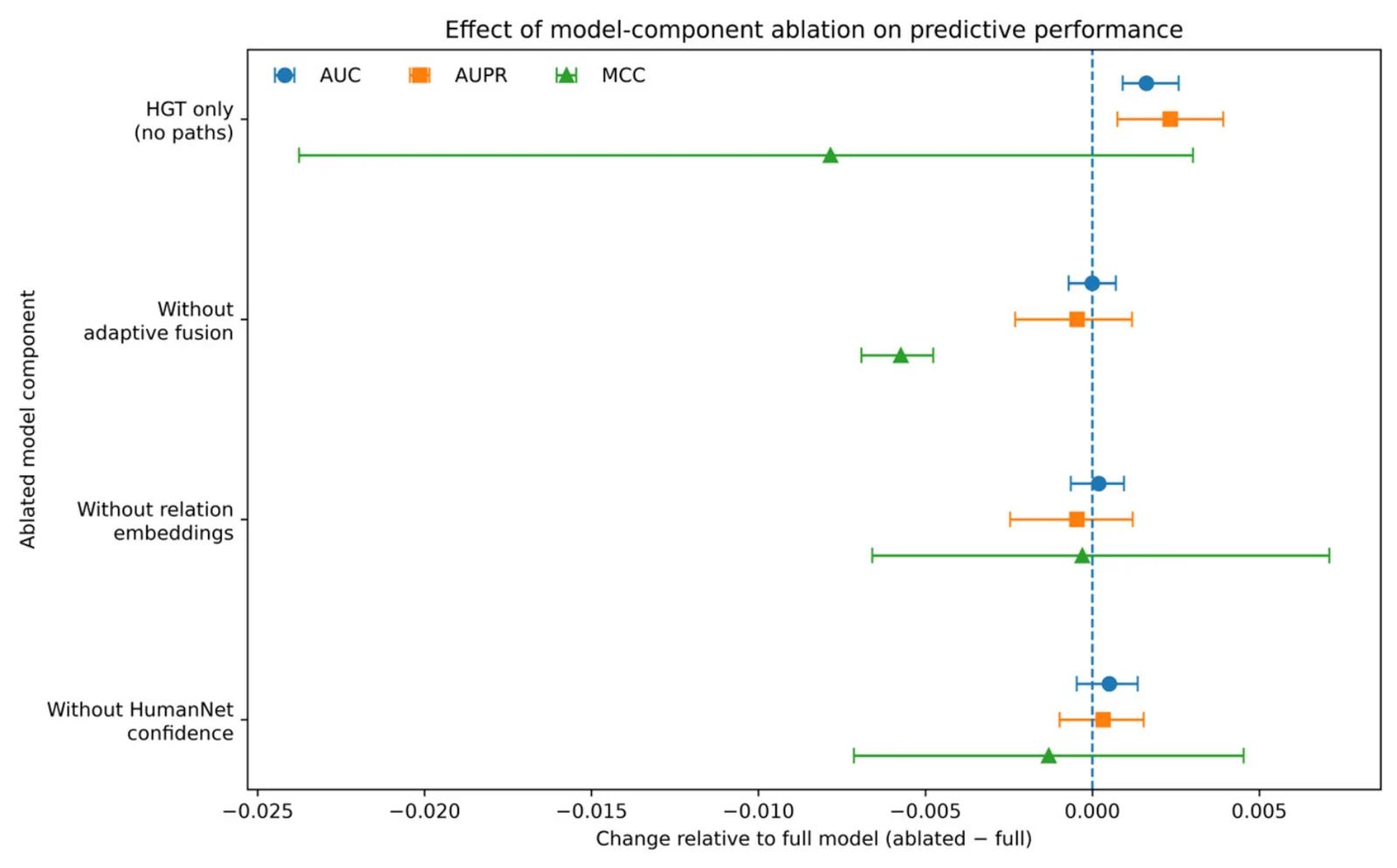
Effect of model-component ablation on predictive performance. Changes in AUC, AUPR, and MCC relative to the full model are shown with 95% bootstrap confidence intervals; negative values indicate reduced performance.

Figure 4 shows the performance change by removing the model components. Most differences in AUC and AUPR were very small and close to zero, which is consistent with the results of Table 6. Although deleting the biological path branch resulted in a slight improvement in AUC and AUPR metrics, this deletion led to lower MCC; thus, the path branch did not help to enhance discrimination on the edge according to AUC and AUPR metrics but may be useful for classification performance at the specific decision threshold. Additionally, the relatively wide confidence intervals, especially for MCC, indicate variability across the five outer folds. On the whole, Figure 4 confirms the conclusion about the major role of the HGT backbone in providing most of the discriminative performance at the edge setting, while the rest of the model elements have a minor effect.

### 2.7. Literature Validation of High-Confidence Predictions

The results of the predictions validated using the literature are summarized in Table 8. Six of the 20 highest-ranked previously unlabeled miRNA-drug pairs were supported by independent studies. There were a number of associations supported by direct functional experiments showing that the miRNA modulated sensitivity or resistance to the predicted drug. The sensitivity to oxaliplatin in resistant colorectal cancer cell lines was enhanced by miR-9, as shown in [16]. There is also evidence in the literature that miR-454-3p promoted oxaliplatin resistance by PTEN/AKT signaling and its inhibition restored sensitivity to oxaliplatin [17]. miR-142-3p is also associated with increased sensitivity to paclitaxel in resistant breast cancer cells [18] and gemcitabine resistance in pancreatic cancer cells [19]. miR-18a-5p was also linked to oxaliplatin sensitivity through the LINC02362/miR-18a-5p/FDX1 pathway [20] and miR-92a-3p was found to be highly abundant in exosomes of gemcitabine-treated CAFs in the presence of a PTEN-related resistance mechanism [21]. Compared to 1 out of the 120 randomly selected unlabeled control pairs (0.83%), 6 of the 20 highest-ranking predictions (30%) had positive literature support.

**Figure 5:**
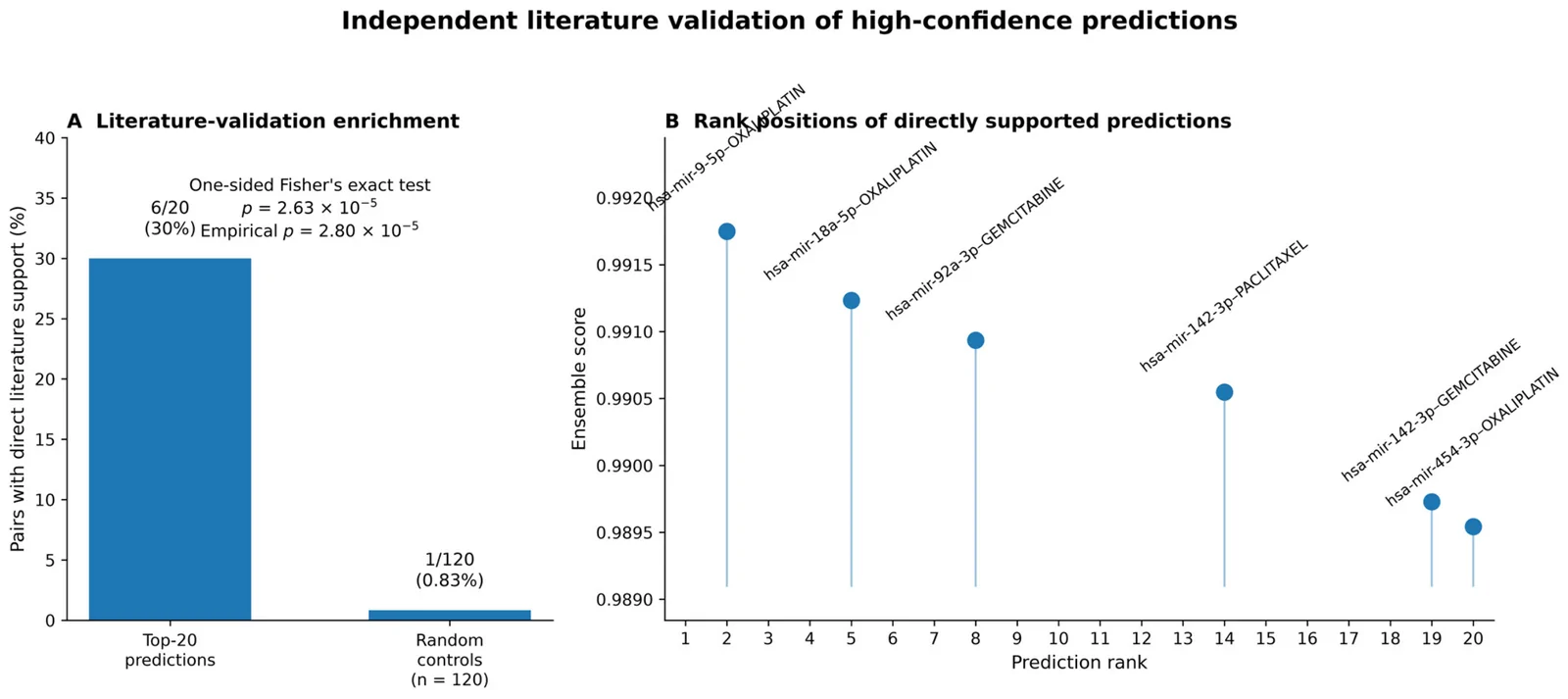
Independent literature validation of high-confidence miRNA–drug predictions. (**A**) Direct literature support was identified for 6 of the 20 highest-ranked previously unlabeled predictions (30%), compared with 1 of 120 randomly selected unlabeled control pairs (0.83%). The difference was significant using a one-sided Fisher’s exact test (odds ratio = 51.0, *p* = 2.63 × 10^−5^). A repeated random-sampling analysis using 1,000,000 random samples of 20 pairs produced an empirical *p* = 2.80 × 10^−5^. (**B**) Rank positions and ensemble scores of the six directly supported predictions within the predefined top-20 set.

As shown in Figure 5, the independent literature assessment identified support for 6 of the 20 highest-ranked previously unlabeled miRNA-drug pairs (30%). As a result, of 120 randomly selected unlabeled control pairs, 1 pair (0.83%) had qualified literature support. The difference was statistically significant by one-sided Fisher’s exact test (odds ratio = 51.0, *p* = 2.63 × 10^−5^). In order to examine whether this enrichment was resilient to the random selection of control pairs, we created 1,000,000 samples of 20 pairs from 140 literature-assessed pairs. Only 27 (out of the 1,000,000) random samples had six or more literature-supported pairs, and *p* = 2.80 × 10^−5^. These results show that the top-ranked predictions in our model had more literature support than randomly selected unlabeled pairs. The literature supported the miRNA–drug associations, but the biological paths shown in Table 8 were model-generated and were not independently verified in the literature.

### 2.8. Prediction-Level Interpretability of Biological Paths

Prediction-level interpretability is performed on the six miRNA-drug associations in Table 8, which are supported by literature evidence based on the three discovery models. The fusion weights that have been learned for the global HGT and biological path representations, the attention weight of the representative biological path, and the change in prediction scores when all biological paths have been masked for the particular pair were obtained. The same trained models produced the predictions in Table 8. The reproduced ensemble probabilities differed from the original values by only approximately 10^−8^–10^−7^.

In accordance with Table 9, the average fusion weight obtained for the biological path representation was 0.599121–0.606766, while the global HGT weight was 0.393234–0.400879. The representative paths were drug-transporter and drug-enzyme relations, where the attention weights were 0.082094–1.000000. The differences in attention are due to the number of available paths; for example, miR-18a-5p-oxaliplatin had only one biological path, and therefore it received an attention weight of 1.000000, while miR-92a-3p-gemcitabine had 16 paths, and its representative path received an attention weight of 0.082094.

Masking all biological paths reduced the mean prediction score for each of the six associations. For miR-9-5p-oxaliplatin, the decrease was 0.008036. For miR-92a-3p-gemcitabine, the decrease was 0.009333, and for miR-142-3p-gemcitabine, it was 0.009326. For miR-18a-5p-oxaliplatin and miR-142-3p-paclitaxel, the decreases were 0.008808 and 0.008770, respectively. For miR-454-3p–oxaliplatin, the decrease was 0.011133. From the results, we can see that the learned biological path representation had a positive effect on all six individual predictions. The perturbation effects were pair-dependent, so the model did not depend primarily on a single drug–gene relation for all predictions.

## 3. Materials and Methods

### 3.1. Dataset and Data Sources

I combined information from complementary public biomedical resources to build a biologically informative heterogeneous network for miRNA–drug response-association prediction. The resulting dataset combines different types of biological and chemical information relevant to miRNA–drug response associations. These types include miRNA expression profiles, miRNA–gene regulatory interactions, drug–gene relationships, gene–gene functional associations, and drug molecular structures. There are three main biological entity types that compose the combined network: miRNAs, drugs, and genes. These entities are linked via different types of relations that suggest different regulatory and pharmacological mechanisms. The miRNA-drug response associations were derived from the ncRNADrug database [7]. This database contains experimental evidence of increased drug resistance and increased drug sensitivity. Based on our preprocessing and the removal of duplicate pairs, we have 5379 unique miRNA–drug pairs. These unique miRNA–drug pairs involve 635 miRNAs and 216 drugs in the final prediction set. A positive label indicates that the miRNA–drug response association is experimentally supported rather than indicating the direction of the response. Based on the 10,491 ncRNADrug source records that were mapped to those final pairs, 4908 were resistant and 5583 were sensitive. At the pair level, 2121 pairs contained only resistance records, 2151 contained only sensitivity records, and 1107 contained both resistance and sensitivity records. The 1107 mixed-direction pairs include 214 pairs that had opposing effects within the same normalized species, phenotype, and condition context, while the remaining 893 pairs had opposing effects across different contexts. Thus, response direction and biological context were not treated as the prediction targets in this study. To identify the miRNA–gene regulatory interactions, the ENCORI (starBase) database was used [22]. ENCORI is a large-scale RNA interaction database that integrates experimental data, particularly CLIP-seq data, to characterize RNA-mediated regulatory interactions. After normalizing the identifiers and filtering them, 27,225 interactions between miRNAs and genes were kept for inclusion in the global network. These interactions provide biologically relevant links between miRNAs and their downstream gene targets. They also allow the model to capture gene-mediated mechanisms that might play a role in drug resistance.

Drug–gene interactions were taken from DrugBank 6.0 [13]. Such drug–gene interactions were annotated so that not all interactions could be described by the same generic relation. I studied four types of drug–gene relations: drug–target, drug–enzyme, drug–transporter, and drug–carrier. Preprocessing steps were carried out and included mapping the drug identifiers, harmonizing gene identifiers, filtering, and removing duplicates. After preprocessing, the final heterogeneous graph was composed of 880 drug–target, 700 drug–enzyme, 418 drug–transporter, and 102 drug–carrier interactions. These DrugBank relationships have different relation types (target, enzyme, transporter, and carrier), and the relation types were retained as separate drug–gene interactions rather than being merged into a single drug–gene interaction.

The human functional gene network was derived from various genomic and proteomic evidence sources. Functional gene–gene associations were obtained from HumanNet [23]. After preprocessing and restriction to genes present in the integrated heterogeneous network, 101,234 gene–gene edges were obtained. These interactions form an additional biological level by which regulatory signals coming from miRNAs can spread towards drug action–related genes and resistance mechanism-related genes. Furthermore, molecular information was added as node-level features along with graph connectivity. Information on miRNA expression profiles was collected from the Cancer Cell Line Encyclopedia (CCLE) [24] and aligned with the miRNAs in the heterogeneous network. The initial miRNA node features of this network were its expression profiles, which provided information complementary to topology. For the drug nodes, SMILES representations of molecular structures were obtained from PubChem [25]. To get structure-aware drug features, the SMILES strings were encoded using a trained ChemBERTa molecular language model, which generates a dense molecular embedding. This output is used as the initial drug-node representation. As a result, the drug representations include information on chemical structure that is independent of the biological connections in the heterogeneous network. The final dataset, constructed after cross-source integration, identifier harmonization, filtering and duplicate removal, consisted of 635 miRNAs, 216 drugs and 3898 genes. Table 10 shows the data sources and the final entities, as well as the biological relations that are used in this study.

### 3.2. Data Integration and Heterogeneous Graph Construction

After collecting the individual data sources, identifiers were harmonized across datasets to build a unified heterogeneous biomedical network. Drug identifiers were matched from the three databases (ncRNADrug, DrugBank, and PubChem) and gene identifiers were normalized prior to integration of ENCORI, DrugBank and HumanNet interactions [13,22,23]. miRNA identifiers were standardized using a human miRNA naming scheme. The redundant associations created in cross-database integration were eliminated.

The resulting heterogeneous graph can be defined as

*G* = (*V*, *E*),

where the node set consists of three entity types,

*V* = *V*_miRNA_ ∪ *V*_drug_ ∪ *V*_gene_.

There are four functionally distinct drug–gene relationships, as well as miRNA–gene and gene–gene relationships, in the biological edge set. This can be expressed as follows:

*E*_bio_ = *E*_miRNA−gene_ ∪ *E*_gene−gene_ ∪ *E*_drug−target_ ∪ *E*_drug−enzyme_ ∪ *E*_drug−transporter_ ∪ *E*_drug−carrier_.

A bidirectional link (relationship) is established when it is needed to pass a message from one end to the other in the graph consisting of different types of nodes. It should be noted that the four types of relationships defined in DrugBank are represented as four separate edge types (as opposed to the drug–gene relationship being represented as a single type of relation), which enables the proper preservation of the relation’s biological semantics when passing a heterogeneous message.

A major difference was drawn between the biological context edges and miRNA–drug response associations used as prediction targets. The entire set of 5379 known associations was therefore not included in the permanent biological graph. Associations between miRNA and drugs representing response associations were only included in the message-passing graph via the current inner training partition. The graph employed for training the model in fold was

$$G_{f}=\left(V,\;E_{\mathrm{bio}}\cup \;E_{\mathrm{MiDrug}}^{\mathrm{train},f}\right).$$

The validation and test partitions of miRNA–drug associations, along with their reverse relations, were excluded from the graph. Hence,

$$E_{\mathrm{MiDrug}}^{\mathrm{val},f}\cap E\left(G_{f}\right)=\emptyset ,$$

And

$$E_{\mathrm{MiDrug}}^{\mathrm{test},f}\cap E\left(G_{f}\right)=\emptyset .$$

By reconstructing the graph on a fold-specific basis, we prevent leakage of target-edge information during message passing and ensure that held-out miRNA–drug response associations are evaluated without any direct link to their labels [8,11]. The biological paths used by the path-based component are constructed using the auxiliary miRNA–gene, gene–gene, and drug–gene relations. Validation and test miRNA–drug response associations were not used for path construction.

### 3.3. Node Feature Representation

Feature vectors corresponding to biological or molecular information about each type of node were used to represent the three node types. The features for the nodes corresponding to miRNAs, drugs and genes were built independently first, and then merged to form the heterogeneous graph.

#### 3.3.1. miRNA Expression Features

A set of expression profiles from the Cancer Cell Line Encyclopedia (CCLE) was used to represent miRNA nodes [24,26]. In the pre-processing step, only human miRNAs with the hsa- prefix were kept, miRNA columns that were duplicated were deleted, and miRNA identifiers were standardized for alignment with other data sources. For the last graph construction, miRNAs were selected only if they appeared in the expression matrix and the integrated set of miRNAs. The expression matrix was transposed, and non-finite values were replaced with zero, so that the profile of each miRNA was represented based on the expression profile of 954 cell lines. There was no further normalization or transformation in the last step of graph construction. This led to an miRNA feature matrix being set as

***X**^m^* ∈ ℝ^635×954^,

Each row is an miRNA, and each column is an expression level in a specific cell-line context. The expression profiles give node-level data that characterizes the difference in miRNA activity in different cell contexts.

#### 3.3.2. Drug Molecular Features

Molecular embeddings derived from chemical structure were used to represent drug nodes. SMILES representations retrieved from PubChem were encoded using the pretrained seyonec/ChemBERTa-zinc-base-v1 checkpoint. ChemBERTa was used as a fixed feature extractor and was not fine-tuned during model training. The model was run in evaluation mode without gradient computation, with a maximum sequence length of 512. The 768-dimensional representation of each drug was obtained by attention-mask-aware mean pooling over the last hidden-state token embeddings. Therefore, the drug feature matrix was determined to be:

***X**^d^* ∈ ℝ^216×768^.

These molecular representations give structural information about the drugs, which is complementary to the heterogeneous network of pharmacological drug–gene interactions.

#### 3.3.3. Gene Features

Auxiliary biological information from the integrated network was used to assemble a high-dimensional sparse feature vector for each gene. The feature blocks were the gene–miRNA incidence matrix, the DrugBank target and enzyme relations matrices, and the transporter and carrier relations matrices, and the HumanNet weighted degree of every gene [23]. No HumanNet confidence-score threshold was applied; HumanNet interactions were only kept for the retained set of the integrated genes; self-interactions between the same gene were eliminated and the maximum possible confidence score was used for interactions between duplicate undirected gene pairs. The composite auxiliary feature matrix was reduced to 200 dimensions by using Truncated Singular Value Decomposition (Truncated SVD) with random_state = 42. The SVD was fitted during construction of the base graph and did not use miRNA–drug response-association labels. This produced the gene feature matrix.

***X**^g^* ∈ ℝ^3898×200^.

This representation captures each gene’s regulatory associations, pharmacological relations, and network connectivity in a reduced-dimensional feature space.

### 3.4. Heterogeneous Graph Transformer Architecture

**Figure 6:**
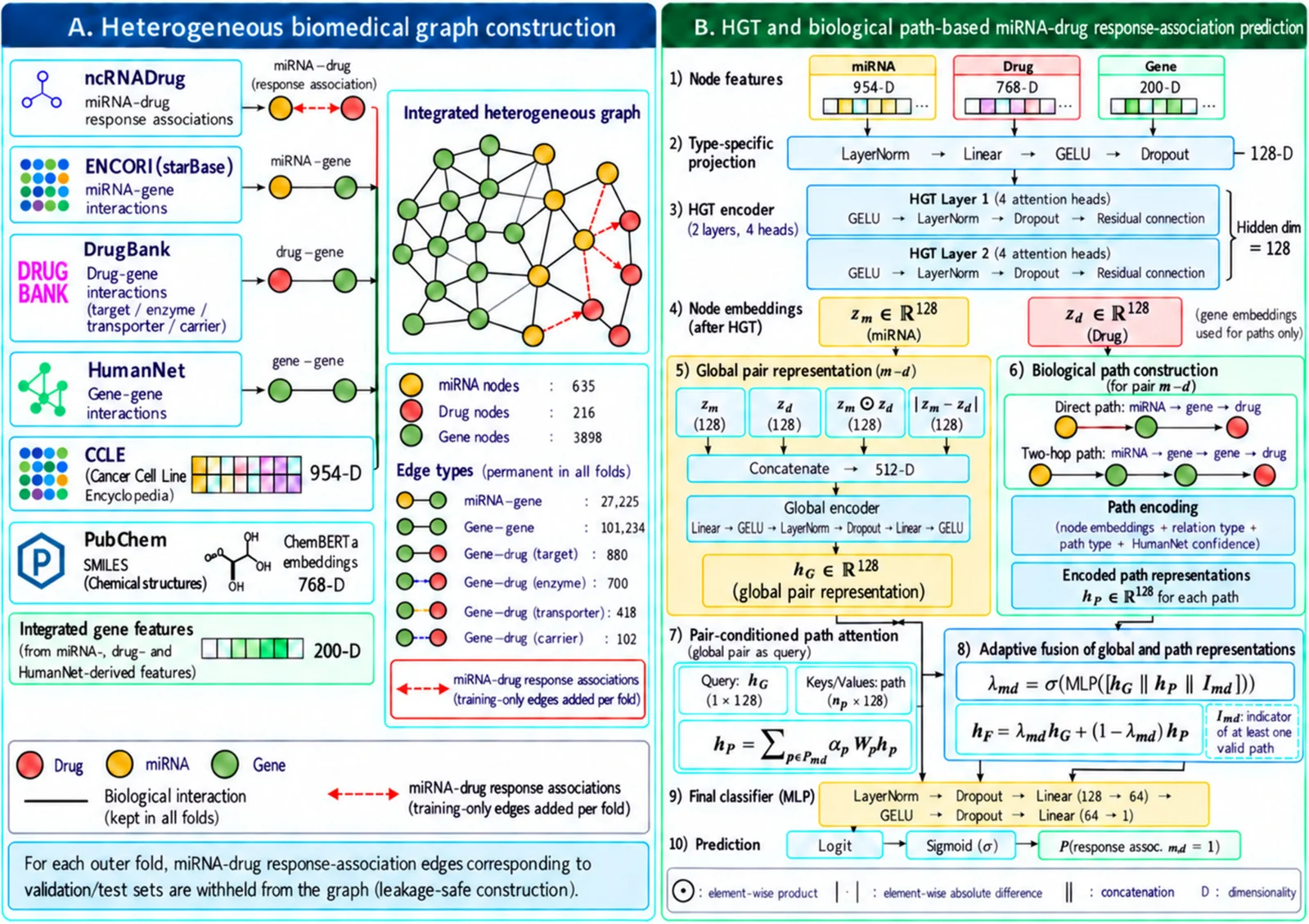
Overview of the heterogeneous biomedical graph and the miRNA–drug response association prediction framework. (**A**) Biological and molecular information from ncRNADrug, ENCORI, DrugBank, HumanNet, CCLE, and PubChem were combined to build the heterogeneous network of miRNA, drug and gene nodes. miRNA–drug response associations were not included in the permanent biological graph. Only the training associations were considered for each fold as target-relation edges, and the validation and test associations were reserved to prevent information leakage. (**B**) The prediction model combines miRNA–drug-specific biological pathways with the graph representation of the HGT for each miRNA–drug pair. The features of nodes were transformed into a common 128-dimensional space and then fed into two relation-aware HGT layers. The embeddings of the miRNAs and drugs were used to create a global pair representation, along with their element-wise product and absolute difference between them. The miRNA–gene–drug and miRNA–gene–gene–drug pathways were encoded and weighted according to their relevance for the candidate pair. The global and path-based representations were integrated by a learned adaptive gate. The fused representation is used as the input for the last classifier to get the probability of an miRNA–drug response association.

A heterogeneous network was used to learn representations of miRNAs, drugs, and genes with a Heterogeneous Graph Transformer (HGT) [14]. This network is an ideal fit for HGT, as it can make use of the differences between nodes and relations when passing messages. This enables the model to process information from genes, drugs and miRNAs according to the biological relations in which they are linked. The model employs information at two levels as illustrated in Figure 6. The layers in the HGT are used to capture the overall structure of the heterogeneous network and to generate a global representation of each candidate miRNA–drug pair. At the same time, the model considers biological pathways, which link the same miRNA and drug via genes. These are direct miRNA–gene–drug paths and longer miRNA–gene–gene–drug paths. Instead of considering all paths equally, the global representation of the candidate pair is employed to identify more relevant paths to make a particular prediction. Finally, a learned gating mechanism is used to combine global and path-based representations before calculating the association probability. This allows the prediction to benefit from the overall network context, in addition to a local network context comprising biological associations between the miRNA and the drug.

#### 3.4.1. Node-Type-Specific Input Projection

The initial feature dimensionalities of the three types of nodes are different: 954 for miRNAs, 768 for drugs and 200 for genes, as described in Section 3.3. Prior to heterogeneous message passing, each node type was thus separately projected to a shared 128-dimensional latent space. The initial representation of a node *v* with type *τ*(*v*) was computed as

$$h_{v}^{\left(0\right)}=\mathrm{Dropout}\left(\mathrm{GELU}\left(W_{\tau \left(v\right)}\mathrm{LN}\left(x_{v}\right)+b_{\tau \left(v\right)}\right)\right),$$

where **x***_v_* is the original node feature vector, LN(.) is the layer normalization function, and **W***_τ_*_(_*_v_*_)_ and **b***_τ_*_(_*_v_*_)_ are learnable parameters for the projection for the specific node type. Thus,

ℝ^954^ → ℝ^128^, for miRNAs,

ℝ^768^ → ℝ^128^, for drugs, and

ℝ^200^ → ℝ^128^, for genes.

This projection is an example of having node-specific transformation parameters while maintaining a common latent dimensionality for the heterogeneous node features. This is the exact same input projection as is used in the final model: LayerNorm → Linear → GELU → Dropout.

#### 3.4.2. Relation-Aware Heterogeneous Message Passing

Finally, the expected node representations were processed with two stacked HGT layers. Four attention heads and 128 hidden dimensionality were used in each of the layers. Information of neighboring nodes is aggregated on a target node *v* via relation-aware multi-head attention. The representation of layer *l* + 1 can be represented conceptually as

$${h\sim }_{v}^{\left(l+1\right)}={\mathrm{HGT}}^{\left(l\right)}\left(h_{v}^{\left(l\right)},\left\{h_{u}^{\left(l\right)}:u\in N\left(v\right)\right\},R\right),$$

The neighborhood of a node *v* in the graph is denoted by 𝒩(*v*) and the relation types between two nodes in the graph are denoted by ℛ. In the HGT operation, the graph metadata are used to identify node and edge types when sending messages. Thus, the messages sent via various biological relationships are not considered as a single homogeneous interaction.

These relations encompass miRNA–gene, gene–gene, drug-target, drug-enzyme, drug-transporter and drug-carrier interactions, along with reverse relationships where applicable, in the full biological graph. The training-only miRNA–drug response relation, as well as the reverse relation, is also added to the fold-specific graph during the model training process. The implementation explicitly establishes the reverse directions for the miRNA–gene and the gene–drug relations and introduces both directions of the training-only miRNA–drug relation.

#### 3.4.3. Residual HGT Representation

After each HGT layer, the node representations were passed through a GELU activation, layer normalization and dropout. Next, a residual link was introduced from the input to the output of the layer [27]:

$$h_{v}^{\left(l+1\right)}=h_{v}^{\left(l\right)}+\mathrm{Dropout}\left[\mathrm{LN}\left(\mathrm{GELU}\left({h\sim }_{v}^{\left(l+1\right)}\right)\right)\right].$$

The residual connections hold information from the previous layer, and the HGT layers incorporate additional heterogeneous neighborhood information. This residual operation is used by the implementation when the dimensionality of the input and the updated representations match. Each representation is mapped to the final 128-dimensional embedding in the final layer of HGT by a node-type-specific linear transformation:

$$z_{v}=W_{\tau \left(v\right)}^{\mathrm{out}}h_{v}^{\left(L\right)}+b_{\tau \left(v\right)}^{\mathrm{out}},$$

where *L* = 2 is the number of HGT layers. Thus, the miRNA, drug and gene representations generated are embedded in the same dimensionality, representing information that flows through their heterogeneous biological neighborhoods.

#### 3.4.4. miRNA–Drug Pair Representation and Prediction

The final HGT embeddings of the miRNA and the drug, ***z**_m_* and ***z**_d_*, respectively, were used to represent the candidate miRNA–drug pair (*m*,*d*) and their interaction. The pair representation can be formulated as follows:

***h**_md_* = [***z**_m_* ‖***z**_d_* ‖(***z**_m_*⊙***z**_d_*)‖|***z**_m_* − ***z**_d_* |],

where ‖ is the concatenation operation and ⊙ is the element-wise multiplication. The first two terms represent individual miRNA and drug representations, and the product and absolute difference terms account for the interaction and difference between the two embeddings. Each embedding is 128 dimensions, and the resulting vector has 512 dimensions. It was then mapped to a 128-dimensional global pair representation, ***h**_G_*. Biological pathways connecting the candidate miRNA and drug are also considered in the model. There were two structures of paths:

*m* → *g* → *d* and *m* → *g*_1_ → *g*_2_ → *d*

The first links an miRNA and a drug via a common gene, while the second adds an intermediate gene–gene connection. The embeddings of the nodes in each path, along with the relation type between drug and gene and the type of the path, were used to represent each path. The HumanNet confidence score was also added to paths with a gene–gene interaction. These features were passed through a path encoder to get a 128-dimensional representation ***h**_p_* for each path.

Labels for miRNA–drug response associations were not added to generate biological paths, which were generated deterministically from the auxiliary biological graph. A maximum of 32 paths were kept for each pair of candidates. A maximum of eight direct miRNA–gene–drug paths were selected, with lower-degree genes preferred in cases of truncation and removal of duplicate miRNA–gene–drug paths. For miRNA–gene–gene–drug paths, HumanNet confidence scores were transformed using and min–max normalized. For each first gene, at most the 20 highest-confidence HumanNet neighbors were considered. The two-gene paths were then ranked primarily by decreasing normalized HumanNet confidence and retained until the overall limit of 32 paths per pair was reached. No random path sampling was used.

Each pair of candidates can have several biological pathways, and each pair may have a variety of pathways. The relevance of the biological paths can vary from one pair to another. Therefore, the model does not simply average them but instead uses the global pair representation to decide on the amount of attention to be given to each path. The query is the global representation ***h**_G_*, and the keys and values are provided by the encoded path. The attention score of path *p* is as follows:

$$e_{p}=\frac{{\left(W_{Q}h_{G}\right)}^{T}\left(W_{K}h_{p}\right)}{\sqrt{D}},$$

Here, ***W**_Q_* and ***W**_K_* represent the learned projection matrices and *D* = 128. These scores are then normalized over the set of paths that are available for the candidate pair in order to get the attention weights:

$$\alpha_{p}=\frac{\exp\left(e_{p}\right)}{\sum_{q\in P_{\mathrm{md}}}\exp\left(e_{q}\right)},$$

where *P_md_* are the set of valid paths between *m* and *d*. The path-level representation can be formulated as:

$$h_{P}=\sum_{p\in P_{\mathrm{md}}}\alpha_{p}W_{V}h_{p},$$

***W**_V_* refers to the learned value projection. Thus, the miRNA–drug pair being evaluated decides on the importance assigned to a path.

A learned gate was used to combine global and path-based representations. For each pair, the gate receives ***h**_G_*, ***h**_P_*, and an indicator *I_md_* indicating whether a valid biological path exists or not:

*λ_md_* = *σ*(*f_gate_*[***h**_G_*‖***h**_P_*‖*I_md_*]).

The fused representation can then be formulated as

***h**_F_* = *λ_md_**h**_G_* + (1 − *λ_md_*)***h**_P_*.

If no valid path is available, the model uses the global HGT representation. Otherwise, the amount of information retrieved from the global graph representation and the amount of information retrieved from the pair-specific biological paths are determined by the gate. Finally, ***h**_F_* was fed into a classifier with layer normalization, dropout, a single hidden layer with 64 hidden units and GELU activation function, and a single output unit. Then, using the sigmoid function, the resulting logit *s_md_* was translated to a probability:

*ŷ_md_* = *σ*(*s_md_*),

where *ŷ_md_* is the estimated probability of the miRNA *m* being associated with the response to drug *d*.

### 3.5. Model Training and Optimization

A binary classification model based on HGT was trained to predict the association between miRNA and drug response. There are no sets of experimentally confirmed negative pairs available along with the positive ones in the available databases. As such, negative samples were created from the absence of a combination of miRNA–drug from the complete set of known positive associations. All known positives were removed from the negative sample regardless of whether they were in the training, validation or test partition. Two negative pairs were sampled for every positive training association. Balanced positive-to-negative ratios were applied to validation and testing. The pairs that were not found in the positive data set were considered unlabeled benchmark examples. The sampled classification evaluation measures discrimination under the defined benchmark distribution and does not reflect the abundance of positive associations in the miRNA and drug candidate space. The AdamW optimizer was used to optimize model parameters, with an initial learning rate of 1 × 10^−4^ and a weight decay of 1 × 10^−4^. Mini-batches of 256 miRNA–drug pairs were used for training and up to a total of 60 epochs were used. The model uses Dropout rate with a value of 0.20. To prevent too large updates, we used gradient clipping with a max norm of 2.0. The learning rate during training was adjusted using ReduceLROnPlateau with a patience of three epochs and a reduction factor of 0.5. To compensate for the imbalance, which was brought by negative sampling, weighted binary cross-entropy with logits was used for training. The positive-class weight was defined as:

$$w_{+}=\frac{N_{-}}{N_{+}}$$

The loss for a pair (*i*,*y_i_*) was defined as follows, where *s_i_* represents the predicted logit

$$L=-\frac{1}{N}\sum_{i=1}^{N}[w_{+}y_{i}\log(\sigma (s_{i}))+(1-y_{i})\log(1-\sigma (s_{i}))]$$

Here, *σ*(⋅) refer to the sigmoid function.

I selected the model hyperparameters based on the edge-based evaluation setting before the final experiment. The tuned parameters are the hidden dimension, number of HGT layers and attention heads, dropout rate, learning rate, and batch size. The values that give the best validation performance are used to adjust the model parameters and then fixed for the edge, miRNA-cold, drug-cold, and double-cold experiments. Accordingly, the cold-start results reflect the transfer of an edge-optimized hyperparameter configuration and should not be interpreted as the best achievable cold-start performance of the architecture. Only the inner-validation partition was used for model selection. Validation AUPR was tracked for every epoch, and early stopping was used if 10 consecutive epochs showed no improvement. The checkpoint with the best validation AUPR was kept. The inner-validation logits were used for temperature scaling after training to calibrate the predicted probabilities [28]. Classification thresholds for threshold-dependent metrics were also computed from the predictions of the inner-validations by maximizing MCC. The kept checkpoint, temperature parameter and threshold were then used for the outer-test partition without further model selection. The outer-test labels were not employed for selection of checkpoints or probability calibration or threshold determinations. Three random seeds (42, 52 and 62) and the same data partition were used to run the experiments. I averaged the calibrated probabilities of these three seeds to calculate the final ensemble predictions.

### 3.6. Evaluation Protocol and Generalization Settings

Experiments were performed under four settings—edge, miRNA-cold, drug-cold, and double-cold—to assess the model at different difficulty levels of generalization. This distinction is crucial, since the conventional random edge split test primarily assesses the prediction of missing associations between entities that might exist in the training data, while the cold start test evaluates the prediction of generalization to entities where no miRNA–drug response associations were present during the training phase [29,30]. A five-fold outer evaluation protocol was used for all evaluation settings. In each outer fold, the test miRNA–drug response associations were completely excluded from target-edge supervision. The outer-training data were further divided into inner-training and inner-validation partitions. The model was fitted using the inner-training associations, while the inner-validation AUPR was used for early stopping and checkpoint selection. The checkpoint with the highest validation AUPR was retained. Inner-validation predictions were also used for probability calibration and threshold selection. The retained model was then evaluated on the held-out outer-test partition without any further training or model selection. Thus, the outer-test data had no role in model fitting, early stopping, checkpoint selection, calibration, or threshold determination. During the test, the biological base graph was maintained as leakage-safe. Not all miRNA–drug response edges were stored on the graph; only the positive training associations were added as target-relation edges during message passing based on the current training partition. No target edges were introduced for validation and test associations. Negative sampling was performed in the same way, with the same split restrictions and excluding all known associations between miRNAs and drugs. The main differences among the four evaluation settings are summarized in Table 11.

#### 3.6.1. Edge-Based Evaluation

The edge setting is the traditional link-prediction scenario. Positive miRNA–drug response associations were not clustered based on the identity of the entities, but by the association level. Thus, if an miRNA or drug is present in the test set, it may be present in other response associations in the training set, and the specific response associations used in the test set were not included. In the inner validation stage, a random sample of the outer-training associations was put aside for validation. The model’s capability of reconstructing unknown response associations between entities that may already be known from other parts of the training graph will be assessed in this setting.

#### 3.6.2. miRNA-Cold Evaluation

The miRNA-cold setting was created to assess generalization to miRNAs that do not have any miRNA–drug response associations in the training set. The miRNAs were divided into outer folds and all response associations involving a held-out test miRNA were placed in the test partition. Consequently, the miRNAs represented in the training and test response associations were disjoint. This restriction was also applied to the selection of the model. For inner model selection, I held out a subset of the outer-training miRNAs for validation. All response associations involving these miRNAs were then excluded from inner-training supervision. Also, as with the positive training pairs, only miRNAs located in the training set were considered for the negative training pairs. Importantly, an unseen miRNA in this setting was not completely removed from the heterogeneous biological network. The specific features of its intrinsic expression and the leakage-free auxiliary biological relations like miRNA–gene interactions remained available. All miRNA–drug response-association supervision for that miRNA was withheld. The experiment thus evaluates the ability of the model to predict response associations from the available biological and feature context of the miRNA without having seen any of its response associations during the training phase.

#### 3.6.3. Drug-Cold Evaluation

The drug-cold setting restricts the drugs to the same extent as the other setting. The drugs were distributed over the outer folds, and all the associations between miRNAs and a drug that was withheld from the training set were assigned to the test partition. There was no response association of an outer-test drug made available for training supervision. Similarly, during inner model selection, the drugs used for validation were removed from the drugs in the outer training set, and early stopping and other model-selection decisions were made based on the unseen-drug condition. In this context, negative training pairs were constructed using drugs that were allowed only in the training phase, while the miRNAs were not held out in the training phase because disjointness is imposed only on drug identities in the drug-cold setting. Similar to the miRNA-cold setting, held-out drugs were given information that was independent of the response-association labels, such as the molecular attributes and leakage-safe biological interactions. In the drug-cold experiment, the model is thus tested with a drug for which no miRNA–drug response association has been observed during training.

#### 3.6.4. Double-Cold Evaluation

The double-cold setting represents the most restrictive generalization scenario. Neither the miRNAs nor the drugs in the test associations contributed any miRNA–drug response associations to training. The training associations were only those between training miRNAs and training drugs so that neither of the two entities in a test pair was in the training response associations. A test association only entered the training set when both the miRNA of the test pair and the drug of the test pair belonged to the same set. The same was done in the inner validation. Only a subset of miRNAs and drugs were held out of the training data for the inner validation, while the training associations were only pairs containing the remaining training miRNAs and drugs. In the double-cold protocol, if we assume that the miRNA sets are given as ℳ*_train_* and ℳ*_test_* and the drugs for the response association are given as 𝒟*_train_* and 𝒟*_test_* then the double-cold protocol satisfies

ℳ*_train_* ∩ ℳ*_test_* = ∅ and 𝒟*_train_* ∩ 𝒟*_test_* = ∅

This separation is specific to the targeted miRNA–drug response relation, as in the other cold-start scenarios. Held-out miRNAs and drugs have their own distinct node properties and additional biological connections. Thus, the double-cold setting tests whether the model can infer an association if neither of the two members of the candidate pair contributes any miRNA–drug response association to training.

In the five outer folds, the double-cold test partitions have 121 held-out miRNAs and 40, 40, 39, 39, and 39 held-out drugs, respectively. The corresponding numbers of positive test associations are 271, 270, 124, 207 and 191, which correspond to 542, 540, 248, 414, and 382 sample pairs after adding the matched unlabeled pairs. Only those with both endpoints being in the same set of held-out miRNAs and drugs should be included in the double-cold test partition. Therefore, those with only a held-out miRNA or only a held-out drug should never be considered as double-cold test pairs. Our retained properties and biological relations mean that this protocol will be used to assess the absence of response association supervision and not to evaluate fully inductive generalization to entirely unseen entities without prior biological information. Generalization to incomplete or unavailable biological connections is still to be established.

#### 3.6.5. Full-Candidate Ranking Evaluation

Apart from the sampled-unlabeled classification benchmark, we also rank candidates for the three cold-start protocols. For miRNA-cold evaluation, every held-out miRNA was scored against all 216 drug nodes. For drug-cold evaluation, each held-out drug was scored against all 635 miRNA nodes. For double-cold evaluation, all combinations between held-out miRNAs and held-out drugs in the corresponding fold were scored, and ranking was also evaluated in both directions. We used known positive associations to compute mean reciprocal rank (MRR), mean average precision (MAP), Hits@1, Hits@5, Hits@10, Hits@20, and Recall@1, Recall@5, Recall@10, and Recall@20. The ranking score was calculated as the mean of the three model seeds of the sigmoid-transformed raw logits, without temperature calibration. The score is considered an association score and is not a calibrated biological probability.

#### 3.6.6. Protocol-Aligned Baseline Models

The three baseline models assessed were identical to the full model with respect to both cold-start partitions and candidate construction. The degree/popularity baseline was not trained, but instead involved assigning scores from the target-association frequencies obtained from the training partition corresponding to it. The feature-only MLP used miRNA expression and the ChemBERTa drug representations. It did not use graph message passing. In the HGT-only model, the pair-specific biological path branch was removed, but the relation-aware HGT backbone was kept. Under drug-cold, double-cold, and miRNA-cold conditions, all three controls were assessed on the same outer-fold partitions as the full model.

### 3.7. Drug-Level Cold-Start Generalization Analysis

To determine the reason for the differences in performance across unseen drugs, I performed a post hoc analysis of the drug-level estimates. Predictions from the drug-cold evaluation were used in this analysis. The AUC for each held-out drug was calculated from the predictions of its grouped miRNA–drug pairs. Then, I compared the resulting AUC with several drug characteristics to find out the factors that were associated with differences in performance. Cosine similarity between the ChemBERTa representation of each held-out drug and those of the response-association-supervised drugs in the corresponding inner-training set was used to measure chemical similarity. For each held-out drug, the highest similarity value was retained. DrugBank target sets were used to assess target similarity. Jaccard similarity was calculated between the target set of each held-out drug and those of the response-association-supervised inner-training drugs, and the highest value was retained when target information was available. Path coverage, mean biological path count, overall drug–gene relation degree, and DrugBank target degree were used to evaluate biological connectivity. Spearman rank correlation was used to evaluate the relationship between each drug characteristic and drug-level AUC. I calculated the confidence intervals for the correlation estimates and applied the Benjamini–Hochberg procedure to account for multiple testing. This analysis was conducted after model evaluation and did not influence model training, model selection, or prediction. Drug-level AUC was calculated only for drugs with both positive and sampled-unlabeled test pairs, resulting in 197 evaluable drugs. As a sensitivity analysis for variation in per-drug support, the correlation analysis was repeated after requiring at least five positive and five sampled-unlabeled pairs per drug.

### 3.8. Generation and Literature Validation of Previously Unlabeled Predictions

High-confidence miRNA–drug pairs that were not part of any known response associations were identified by conducting a separate analysis. This analysis was performed after the cross-validation experiments and is not used to estimate model performance. First, an internal development split was used to determine an appropriate training duration. The fitted model in this step was discarded. A new model was trained from scratch for each random seed to obtain the final prediction results. The training was conducted using all known positive miRNA–drug response associations as supervision.

After training, all miRNA–drug pairs that were not known to have any response association were scored. To obtain an ensemble score for each previously unlabeled pair, I averaged the predictions from the three random seeds, and then the pairs were ranked according to these scores. Prior to the literature assessment, the top 20 highest-ranked previously unlabeled associations were chosen. There was a set of 20 randomly selected pairs of unlabeled items as a control. The top 20 predictions and previously assessed controls were removed, and 100 additional control pairs were then sampled without replacement from the remaining pairs of controls, to alleviate the risk of relying on one or a few random control sets. The PubMed database was queried up to 25 September 2026, with drug name and miRNA name combinations and the terms “response”, “sensitivity” and “resistance” to find independent evidence for each miRNA–drug association. Only evidence from an independent study that reported an experimentally supported association between the miRNA and the associated drug response was regarded as supportive. Where available, direction of the reported association (resistance or sensitivity), and cancer or cellular context were noted. The percentage of literature-supported association predictions for the top 20 predictions was compared to the percentage of literature-supported association predictions for the 120 randomly selected controls with a one-sided Fisher’s exact test. A repeated random-sampling analysis was conducted to determine if the enrichment depended on the specific control selected, using 1,000,000 samples of 20 literature-assessed pairs drawn without replacement from the total of 140 pairs.

The number of random samples with six or more literature-supported associations was used to calculate the empirical *p*-value using the standard +1 correction. The literature-based assessment was considered independent support for the biological relevance of the ranked predictions. The literature evidence was treated as independent support for the miRNA–drug response association, not as evidence for the intermediate genes in the model-derived biological paths. These literature-based assessments were not considered an alternative to prospective experimental validation.

### 3.9. Evaluation Metrics and Statistical Analysis

Area under the curve (AUC), area under precision-recall curve (AUPR), accuracy (ACC), precision, recall, F1-score and Matthews correlation coefficient (MCC) were used to assess model performance. AUC and AUPR were the primary threshold-independent metrics of predictive performance [31,32], whereas ACC, F1-score and MCC were used to evaluate performance on the selected threshold in classification. MCC was added because it considers all four outcomes of the confusion matrix and thus gives a balanced assessment of the performance of a binary classification [33]. Using the validation data, the threshold that maximizes the MCC on the validation predictions was kept and then applied to the outer test predictions. This ensures that the test labels did not influence the selection of thresholds. The Brier score [34] and expected calibration error (ECE) [28] were also used to evaluate the quality of the predicted probabilities. These metrics evaluate how well the probabilities are calibrated, with small values indicating better calibration. Since these calibration measures were obtained on sampled evaluation sets with about balanced levels of positive-to-unlabeled ratios, they reflect calibration within the benchmark distribution. They should not be viewed as estimates of biological association probabilities in the candidate space. The performance of each outer fold was evaluated separately using the ensemble predictions, and the performance metrics across the five outer folds were summarized as mean ± standard deviation and 95% confidence interval.

## 4. Conclusions

In this work, a relation-aware heterogeneous learning framework was developed to predict miRNA-drug response associations. The framework can evaluate whether its predictions can be applied to entities that do not appear in the training response associations. miRNA expression profiles, drug molecular representations, miRNA–gene regulatory interactions, gene–gene functional associations and different drug–gene pharmacological relations were combined in a unified heterogeneous network. The proposed framework model achieved good performance in an edge-based setting. The model maintained its predictive power when miRNAs assigned to the test set were not included in the response associations used in the training process. The performance became worse when the response associations of the test drugs did not appear in the training (drug-cold setting). Also, a decline in the model performance was observed when associations between both the test miRNAs and test drugs were excluded (double-cold setting). This indicates that strong performance (i.e., strong edge-based evaluation) does not mean that the model will perform as well on drugs unseen in the context of response association supervision. The same was true for the drug-level analysis.

Prediction performance in the case of unseen drugs was not significantly associated with ChemBERTa molecular or DrugBank target similarity. Therefore, similarity to drugs in the training data did not significantly explain the generalization of the model to new drugs. The ablation experiments showed that the HGT backbone showed the highest discriminative performance for edge-based evaluation. The biological path and fusion components had no clear impact on performance in the edge-based evaluation, and their effects varied across the evaluation metrics. The presence of biological context in the pair may be more relevant to prediction-level biological interpretation than to improving the standard ranking metrics. Independent literature evaluation supported 6 of the 20 highest-ranked previously unlabeled relationships, compared to 1 of 120 randomly selected controls. Based on these results, we can conclude that candidate miRNA-drug response associations can be prioritized by a relation-aware heterogeneous graph, but the results also reveal an important weakness. In particular, there is no guarantee that a model will generalize well to previously unseen drugs if it has strong edge-level performance. This suggests that the greater challenge is generalization to unseen drugs, and therefore it should be investigated further in future work. Also, more improvements may be needed in molecular-level representations and in the validation of high-confidence predictions. These improvements might involve additional drug-specific pharmacological information beyond molecular and target similarity, as well as experimental validation. Future work could explore more advanced graph learning approaches to learn better representations of miRNAs and drugs. One possible approach is Neighborhood Consensus-guided Reinforcement Learning [35], which can adaptively select node neighborhoods using a task-oriented reward to support neighborhood consensus. Although originally developed for graph clustering, this approach could be adapted to predict miRNA–drug associations by selecting informative heterogeneous neighbors. Another possibility is to use global encoding mechanisms such as RANGE [36] that use attention-based aggregation and broadcast through virtual representations to provide better long-range information transmission and avoid oversquashing. Such mechanisms could be implemented in the HGT backbone to capture complementary local and long-range dependencies in the miRNA–gene-drug graph. To determine if the models can be used to predict response associations beyond those in the training set, future studies should continue to evaluate models when cold-start conditions are taken into account in the analysis.

## Figures and Tables

**Table 1:** Predictive performance of the proposed model across five outer folds under edge-based evaluation.

Metric	Fold 1	Fold 2	Fold 3	Fold 4	Fold 5	Mean ± SD	95% CI
AUC	0.9561	0.9473	0.9582	0.9513	0.9557	0.9537 ± 0.0044	0.9499–0.9576
AUPR	0.9505	0.9395	0.9568	0.9459	0.9543	0.9494 ± 0.0069	0.9433–0.9554
ACC	0.8973	0.8927	0.8871	0.8862	0.8870	0.8900 ± 0.0048	0.8858–0.8943
F1	0.8980	0.8899	0.8869	0.8898	0.8871	0.8904 ± 0.0045	0.8864–0.8943
Precision	0.8918	0.9130	0.8882	0.8622	0.8859	0.8882 ± 0.0181	0.8724–0.9041
Recall	0.9043	0.8680	0.8857	0.9191	0.8884	0.8931 ± 0.0194	0.8761–0.9101
MCC	0.7947	0.7863	0.7742	0.7740	0.7740	0.7806 ± 0.0095	0.7723–0.7889
Brier	0.0816	0.0856	0.0822	0.0847	0.0831	0.0834 ± 0.0017	0.0820–0.0849
ECE	0.0363	0.0368	0.0390	0.0351	0.0299	0.0354 ± 0.0034	0.0325–0.0384

**Table 2:** Predictive performance of the proposed model under cold-start evaluation.

Metric	miRNA-Cold	Drug-Cold	Double-Cold
AUC	0.9466 ± 0.0061	0.6685 ± 0.0354	0.6450 ± 0.0720
AUPR	0.9436 ± 0.0049	0.6534 ± 0.0569	0.6637 ± 0.0884
ACC	0.8812 ± 0.0095	0.6049 ± 0.0606	0.5853 ± 0.0579
F1	0.8829 ± 0.0095	0.4699 ± 0.1884	0.4289 ± 0.2402
Precision	0.8709 ± 0.0136	0.6721 ± 0.1033	0.6451 ± 0.1547
Recall	0.8955 ± 0.0176	0.3916 ± 0.2114	0.3825 ± 0.2719
MCC	0.7631 ± 0.0190	0.2305 ± 0.1275	0.1910 ± 0.1274
Brier	0.0897 ± 0.0062	0.2463 ± 0.0042	0.2393 ± 0.0125
ECE	0.0342 ± 0.0073	0.0823 ± 0.0341	0.0608 ± 0.0262

**Table 3:** Full-candidate ranking performance of the proposed model under miRNA-cold, drug-cold, and double-cold evaluation. Results are reported as mean ± SD across five outer folds. For double-cold evaluation, ranking was assessed in both miRNA-to-drug and drug-to-miRNA directions.

Setting	Direction	MRR	MAP	Hits@10	Recall@10	Hits@20	Recall@20
miRNA-cold	miRNA→drug	0.8044 ± 0.0389	0.5462 ± 0.0216	0.9669 ± 0.0131	0.5861 ± 0.0160	0.9917 ± 0.0058	0.8272 ± 0.0110
drug-cold	drug→miRNA	0.2343 ± 0.0282	0.1348 ± 0.0149	0.4162 ± 0.0648	0.1267 ± 0.0407	0.5228 ± 0.0275	0.2121 ± 0.0400
double-cold	miRNA→drug	0.2400 ± 0.1760	0.1872 ± 0.1213	0.5191 ± 0.3298	0.3285 ± 0.2501	0.7855 ± 0.2081	0.6294 ± 0.2617
double-cold	drug→miRNA	0.3533 ± 0.0328	0.2404 ± 0.0169	0.5713 ± 0.1162	0.2280 ± 0.0518	0.7220 ± 0.0905	0.4058 ± 0.0506

**Table 4:** Protocol-aligned comparison of the full model with degree/popularity, feature-only MLP, and HGT-only controls under cold-start evaluation. Classification AUC and AUPR were calculated using the sampled-unlabeled benchmark, whereas MRR and MAP were calculated using full-candidate ranking. Results are reported as mean ± SD across five outer folds.

Setting	Ranking Direction	Model	AUC	AUPR	MRR	MAP
miRNA-cold	miRNA→drug	Full model	0.9466 ± 0.0061	0.9436 ± 0.0049	0.8044 ± 0.0389	0.5462 ± 0.0216
		Degree/popularity	0.9306 ± 0.0064	0.9234 ± 0.0033	0.8152 ± 0.0365	0.5664 ± 0.0256
		Feature-only MLP	0.9261 ± 0.0053	0.9203 ± 0.0066	0.7988 ± 0.0354	0.4945 ± 0.0206
		HGT-only	0.9468 ± 0.0056	0.9440 ± 0.0043	0.8006 ± 0.0375	0.5449 ± 0.0210
drug-cold	drug→miRNA	Full model	0.6685 ± 0.0354	0.6534 ± 0.0569	0.2343 ± 0.0282	0.1348 ± 0.0149
		Degree/popularity	0.7179 ± 0.0444	0.6833 ± 0.0421	0.2967 ± 0.0324	0.1768 ± 0.0225
		Feature-only MLP	0.6300 ± 0.0772	0.5975 ± 0.0828	0.2751 ± 0.0148	0.1451 ± 0.0246
		HGT-only	0.6470 ± 0.0202	0.6091 ± 0.0308	0.2663 ± 0.0369	0.1442 ± 0.0292
double-cold	miRNA→drug	Full model	0.6450 ± 0.0720	0.6637 ± 0.0884	0.2400 ± 0.1760	0.1872 ± 0.1213
		Degree/popularity	0.5000 ± 0.0000	0.5000 ± 0.0000	0.1732 ± 0.1709	0.1364 ± 0.0944
		Feature-only MLP	0.6401 ± 0.0634	0.6136 ± 0.0695	0.1858 ± 0.0299	0.1603 ± 0.0182
		HGT-only	0.6224 ± 0.0627	0.6194 ± 0.0787	0.1566 ± 0.0869	0.1332 ± 0.0556
double-cold	drug→miRNA	Full model	0.6450 ± 0.0720	0.6637 ± 0.0884	0.3533 ± 0.0328	0.2404 ± 0.0169
		Degree/popularity	0.5000 ± 0.0000	0.5000 ± 0.0000	0.2474 ± 0.1293	0.1515 ± 0.0466
		Feature-only MLP	0.6401 ± 0.0634	0.6136 ± 0.0695	0.2794 ± 0.0591	0.2136 ± 0.0224
		HGT-only	0.6224 ± 0.0627	0.6194 ± 0.0787	0.3949 ± 0.1250	0.2469 ± 0.0259

**Table 5:** Associations between drug-level characteristics and predictive performance under drug-cold evaluation.

Drug-Level Characteristic	*N*	Spearman ρ	95% CI	*p*-Value	BH-Adjusted q
Biological path coverage	197	−0.234	−0.359 to −0.103	0.0009	0.0092
Mean number of biological paths	197	−0.232	−0.361 to −0.098	0.0010	0.0092
Drug–gene relation degree	197	−0.181	−0.314 to −0.036	0.0111	0.0445
DrugBank target degree	197	−0.166	−0.307 to −0.023	0.0199	0.0488
Maximum ChemBERTa similarity	197	0.015	−0.137 to 0.157	0.8320	0.8320
Maximum target Jaccard similarity	145	−0.046	−0.210 to 0.106	0.5804	0.7065

Note: the table reports the analysis across all evaluable drugs; a minimum-support sensitivity analysis is reported in the accompanying text. *N* represents the number of drugs included in each correlation analysis.

**Table 6:** Ablation analysis under edge-based evaluation. Results are reported as mean ± SD across five outer folds using the same data partitions and training settings.

Model Variant	AUC	AUPR	ACC	F1	MCC	Brier
Full model	0.9537 ± 0.0044	0.9494 ± 0.0069	0.8900 ± 0.0048	0.8904 ± 0.0045	0.7806 ± 0.0095	0.0834 ± 0.0017
HGT only (no paths)	0.9553 ± 0.0037	0.9517 ± 0.0066	0.8845 ± 0.0070	0.8889 ± 0.0038	0.7728 ± 0.0097	0.0820 ± 0.0015
Without adaptive fusion	0.9537 ± 0.0045	0.9489 ± 0.0072	0.8868 ± 0.0058	0.8880 ± 0.0051	0.7749 ± 0.0102	0.0835 ± 0.0026
Without relation embeddings	0.9539 ± 0.0041	0.9489 ± 0.0071	0.8900 ± 0.0059	0.8906 ± 0.0067	0.7803 ± 0.0118	0.0832 ± 0.0016
Without HumanNet confidence	0.9542 ± 0.0034	0.9497 ± 0.0055	0.8896 ± 0.0051	0.8895 ± 0.0046	0.7793 ± 0.0102	0.0828 ± 0.0015

**Table 7:** Cold-start ablation analysis under drug-cold and double-cold evaluation. AUC and AUPR were calculated using the sampled-unlabeled classification benchmark, whereas MRR and MAP were calculated using full-candidate ranking. Results are reported as mean ± SD across five outer folds.

Setting	Ranking Direction	Model Variant	AUC	AUPR	MRR	MAP
Drug-cold	drug→miRNA	Full model	0.6762 ± 0.0442	0.6710 ± 0.0580	0.2343 ± 0.0282	0.1348 ± 0.0149
		HGT-only	0.6470 ± 0.0202	0.6091 ± 0.0308	0.2663 ± 0.0369	0.1442 ± 0.0292
		Fixed fusion	0.6812 ± 0.0455	0.6747 ± 0.0594	0.2307 ± 0.0289	0.1284 ± 0.0102
		Relation-collapsed	0.6279 ± 0.0470	0.6026 ± 0.0466	0.2440 ± 0.0221	0.1407 ± 0.0181
		No ChemBERTa	0.6292 ± 0.0542	0.5961 ± 0.0517	0.2239 ± 0.0411	0.1351 ± 0.0254
Double-cold	miRNA→drug	Full model	0.6392 ± 0.0780	0.6591 ± 0.0967	0.2400 ± 0.1760	0.1872 ± 0.1213
		HGT-only	0.6224 ± 0.0627	0.6194 ± 0.0787	0.1566 ± 0.0869	0.1332 ± 0.0556
		Relation-collapsed	0.6182 ± 0.0642	0.6361 ± 0.0826	0.1890 ± 0.1046	0.1408 ± 0.0613
		No ChemBERTa	0.5944 ± 0.0366	0.5849 ± 0.0490	0.1186 ± 0.0562	0.1201 ± 0.0535
Double-cold	drug→miRNA	Full model	0.6392 ± 0.0780	0.6591 ± 0.0967	0.3533 ± 0.0328	0.2404 ± 0.0169
		HGT-only	0.6224 ± 0.0627	0.6194 ± 0.0787	0.3949 ± 0.1250	0.2469 ± 0.0259
		Relation-collapsed	0.6182 ± 0.0642	0.6361 ± 0.0826	0.3645 ± 0.0565	0.2557 ± 0.0195
		No ChemBERTa	0.5944 ± 0.0366	0.5849 ± 0.0490	0.3452 ± 0.0602	0.2353 ± 0.0231

Note: HGT-only removes the pair-specific biological path branch. Fixed fusion replaces adaptive fusion with equal 0.5/0.5 weighting of the global HGT and path representations. Relation-collapsed merges the DrugBank target, enzyme, transporter, and carrier relations into a single generic drug–gene relation. No ChemBERTa sets the drug molecular feature vectors to zero. Fixed fusion was evaluated only under the drug-cold setting.

**Table 8:** Literature-supported high-confidence predictions among previously unlabeled miRNA–drug pairs and their representative model-derived biological pathways.

Predicted Association	Ensemble Score	Independent Experimental Evidence	Representative Model-Derived Path
miR-9-5p–oxaliplatin	0.9917	Functional: miR-9 overexpression increased oxaliplatin sensitivity in resistant colorectal cancer cells through the lncRNA-p21/miR-9/SIRT1 regulatory axis [16].	miR-9-5p → POU2F1 → SLC22A2 → oxaliplatin
miR-18a-5p–oxaliplatin	0.9912	Functional/mechanistic: the LINC02362/miR-18a-5p/FDX1 axis was experimentally linked to oxaliplatin sensitivity in hepatocellular carcinoma cells [20].	miR-18a-5p → PHF19 → NQO1 → oxaliplatin
miR-92a-3p–gemcitabine	0.9909	Mechanistic: gemcitabine increased exosomal miR-92a-3p released by cancer-associated fibroblasts as part of a PTEN-related chemoresistance mechanism in pancreatic cancer [21].	miR-92a-3p → SPRYD4 → SLC29A2 → gemcitabine
miR-142-3p–paclitaxel	0.9905	Functional: miR-142-3p increased paclitaxel sensitivity in resistant breast cancer cells through the GNB2–AKT–mTOR pathway [18].	miR-142-3p → CAPZA2 → ABCC1 → paclitaxel
miR-142-3p–gemcitabine	0.9897	Functional/mechanistic: the SBF2-AS1/miR-142-3p/TWF1 axis was experimentally implicated in gemcitabine resistance in pancreatic cancer cells [19].	miR-142-3p → TAOK1 → SLC29A2 → gemcitabine
miR-454-3p–oxaliplatin	0.9895	Functional: miR-454-3p promoted oxaliplatin resistance, whereas its inhibition restored oxaliplatin sensitivity in resistant colorectal cancer cells through PTEN/AKT signaling [17].	miR-454-3p → CANX → ABCC2 → oxaliplatin

Note: The independent literature evidence supports the corresponding miRNA–drug response association. The representative biological pathways are model-derived hypotheses and the intermediate genes in these pathways were not independently validated by the cited studies.

**Table 9:** Prediction-level interpretability of the six literature-supported miRNA-drug associations. The weighted average of the fusion weights, attention values, and masking effects for the three saved discovery models are shown. The drug–gene relation is the highest-ranking biological path for each association. Δ score is calculated as the full model prediction score minus the score obtained after masking all biological paths. Positive values indicate a reduction in prediction score after removing biological paths.

miRNA–Drug Association	No. of Paths	Representative Relation	Path Fusion Weight	Representative-Path Attention	Δ Score After All-Path Masking
miR-9-5p–oxaliplatin	4	Drug-transporter	0.599448	0.280320	0.008036
miR-18a-5p–oxaliplatin	1	Drug-enzyme	0.606766	1.000000	0.008808
miR-92a-3p–gemcitabine	16	Drug-transporter	0.599121	0.082094	0.009333
miR-142-3p–paclitaxel	6	Drug-transporter	0.602347	0.217599	0.008770
miR-142-3p–gemcitabine	4	Drug-transporter	0.601714	0.259537	0.009326
miR-454-3p–oxaliplatin	7	Drug-transporter	0.605916	0.158831	0.011133

**Table 10:** Summary of the integrated dataset used for miRNA–drug response association prediction.

Data Component	Source	Role in the Framework	Final Count
miRNA nodes	ncRNADrug/CCLE	Regulatory entities	635
Drug nodes	ncRNADrug/DrugBank/PubChem	Drug entities	216
Gene nodes	ENCORI/DrugBank/HumanNet	Intermediate biological entities	3898
miRNA–drug response association	ncRNADrug	Prediction targets	5379
miRNA–gene	ENCORI	Regulatory interaction	27,225
Gene–gene	HumanNet	Functional interaction	101,234
Drug–target	DrugBank	Pharmacological relation	880
Drug–enzyme	DrugBank	Pharmacological relation	700
Drug–transporter	DrugBank	Pharmacological relation	418
Drug–carrier	DrugBank	Pharmacological relation	102
miRNA expression	CCLE	miRNA node features	635 miRNAs
Drug molecular structure	PubChem	SMILES-derived drug features	216 drugs

**Table 11:** The evaluation settings employed to evaluate miRNA–drug response association prediction with increasing levels of cold-start generalization. The “seen” and “unseen” concern the involvement in miRNA–drug response associations for training, while intrinsic node features and leakage-safe auxiliary biological relations are kept in the cold-start settings.

Setting	Test miRNA Seen in Training Response Associations?	Test Drug Seen in Training Response Associations?	Auxiliary Features and Biological Relations Retained?
Edge	Possible	Possible	Yes
miRNA-cold	No	Possible	Yes
Drug-cold	Possible	No	Yes
Double-cold	No	No	Yes

## Data Availability

The datasets generated and analyzed during the current study are available in the GitHub repository at https://github.com/murtadaBashir/RAHGL-MDR (accessed on 31 August 2026).
